# Job satisfaction and intention to quit: an empirical analysis of nurses in Turkey

**DOI:** 10.7717/peerj.1896

**Published:** 2016-04-26

**Authors:** Abdul Kadar Muhammad Masum, Md. Abul Kalam Azad, Kazi Enamul Hoque, Loo-See Beh, Peter Wanke, Özgün Arslan

**Affiliations:** 1 Department of Administrative Studies & Politics, University of Malaya, , Malaysia; 2 Department of Applied Statistics, Faculty of Economics and Administration, University of Malaya, Kuala Lumpur, Malaysia; 3 Department of Educational Management, Planning and Policy/Faculty of Education, University of Malaya, Kuala Lumpur, Malaysia; 4 Department of Administrative Studies and Politics, Faculty of Economics and Administration, University of Malaya, Kuala Lumpur, Malaysia; 5 COPPEAD Graduate Business School, Federal University of Rio de Janeiro, Rua Paschoal Lemme, Rio de Janeiro, Brazil; 6 Department of Nursing, Faculty of Health Sciences, Marmara University, İstanbul, Turkey

**Keywords:** Job satisfaction, Nursing, Nurse, Intention to quit, Turkey

## Abstract

The aim of this study was to identify the facets influencing job satisfaction and intention to quit of nurses employed in Turkey. Using a non-probability sampling technique, 417 nurses from six large private hospitals were surveyed from March 2014 to June 2014. The nurses’ demographic data, their job-related satisfaction and turnover intentions were recorded through a self-administered questionnaire. In this study, descriptive and bivariate analyses were used to explore data, and multivariate analysis was performed using logistic regression. Nurses’ job satisfaction was found at a moderate level with 61% of the nurses intended to quit. Nevertheless, nurses reported a high satisfaction level with work environment, supervisor support, and co-workers among the selected nine facets of job satisfaction. They also reported a low satisfaction level with contingent reward, fringe benefits, and pay. The impact of demographic characteristics on job satisfaction and intention to quit was also examined. The study revealed a negative relationship between job satisfaction and intention to quit the existing employment. Moreover, satisfaction with supervisor support was the only facet that significantly explained turnover intent when controlling for gender, age, marital status, education, and experience. The implications for nurse management were also described for increasing nurses’ job satisfaction and retention. This study is beneficial for hospital management to ensure proper nursing care that would lead to a better quality healthcare service.

## Introduction

Globally, the present shortage of nurses is a problematic agenda among the health care sectors. Consequently, the quality of patient care services has decreased (Van Bogaert et al., 2010). Thus, ensuring a high level of job satisfaction among nurses and taking proper precautions to avoid their intention to quit are prime concerns (Sabanciogullari & Dogan, 2015). The European Commission expected that by the year 2020 there will be a scarcity of 590,000 nurses in Europe (Flinkman, Isopahkala-Bouret & Salanterä, 2013). According to a recent report from OECD (Organization for Economic Co-operation and Development), there are 16.6 nurses for 1,000 persons in Switzerland, 10.1 in Australia, 8.8 in the OECD countries, 8.6 in UK, and only 1.7 in Turkey (OECD, 2013). In contemporary studies, researchers acknowledged that the quality of patient care is allied with higher staffing levels in nursing. Also, patient satisfaction for healthcare service is related to nurses’ job satisfaction (Flinkman, Leino-Kilpi & Salanterä, 2010; Hyrkas & Morton, 2013; Meeusen et al., 2011). Moreover, nurses’ higher levels of dissatisfaction leads to turnover from the nursing profession (Banaszak-Holl & Hines, 1996), which generally creates staff shortage, increases overtime and job stress, increases burnout, longer patient waiting lists, and finally, as a consequence increased displeasure among patients. The turnover of nurses also increases recruitment and orientation costs (Murrells, Robinson & Griffiths, 2008).

Job satisfaction is the level of serenity that someone feels for work, and this feeling influences performance. In the case of nurses’ job satisfaction, earlier research has revealed that it is negatively connected with nurses’ intention to quit the workplace as well as their nursing profession (Applebaum et al., 2010; Cowin et al., 2008). Hence, proper understanding of factors of nurses’ job satisfaction is essential for proactive managers to take necessary actions. Scholars identified individual characteristics, work requirements and policies, professional status, pay, working environment, coworker support, and administrative style as important factors that influencing nurse’ job satisfaction (Han & Jekel, 2011). Given the past review of literature, it is explicit that there are additional influential job satisfaction factors for nursing. These are age, gender, marital status, organization itself, employment type, work duration, level of responsibility, payments, financial benefits, and recognition (Kacel, Miller & Norris, 2005; Mrayyan, 2005). Moreover, Heinen et al. (2013) acknowledged that characteristics of the work setting, professional identity, job satisfaction, and burnout are the most persuasive factors that influence nurses’ decision to remain or to quit their job. Earlier research shows that turnover in nursing is a logical consequence of nurses’ job dissatisfaction. Initially, nurses quit the unit, then the hospital, and lastly the occupation (Iliopoulou & While, 2010).

In the Turkish healthcare system, there is an acute shortage of nurses owing to extended working hours, inadequate opportunities of on the job training, dearth of chances to work autonomously, and deficiency of care from their superintendents (Gök & Kocaman, 2011; Top & Gider, 2013). In addition, patient load has adverse impact on work settings and the psychological or/and physical health of the Turkish nurses (Yurumezoglu & Kocaman, 2016). Given these situations, in order to enhance strategic managerial decision and expansion growth of hospitals, it is of paramount importance that the factors of nurses’ job satisfaction and turnover intentions be studied to improve their service quality. To the best of our knowledge, there is a dearth of such study on nurses in Turkey. The main thrust of this study was to scrutinize the level of job satisfaction of nurses, and to investigate the effects of socio-demographic characteristics on job satisfaction of nurses’ employed in Turkish private hospitals. In addition, nurses’ intention to quit from existing job position was also investigated in this study.

This research makes two contributions to the literature. Firstly, the finding that the model of intention to quit built upon theories and empirical evidence also applies to Turkey’s health care system. Here, the logistic regression model of intention to quit can be generalized to the overall system. Secondly, the findings of the present study that explore significant predictors of nurses’ intention to quit include the six explanatory variables (i.e., promoting, fringe benefits, contingent rewards, ages and experience). These findings provide strong evidence for the importance of these six significant variables in explaining intention to quit in the context of Turkey. Previous literature shows a consensus that decreases in intention to quit levels will raise the quality of the system. Thus, the findings on factors that contribute to nurses’ job satisfaction and their intention to quit will provide useful knowledge and importance to nursing management and leadership. This would be crucially and considerably advantageous in health care HR planning processes in Turkey. Moreover, the developed regression model provides insights to future researchers on the enhancement of nurses’ job satisfaction and service quality of hospitals in Turkey and other emerging nations.

## Overview of the Literature

In this section, we briefly describe the factors of job satisfaction, job dissatisfaction and intention to quit of nurses from the present workplace. Some keywords are examined including nurses, nursing, job dissatisfaction, job satisfaction, intention to quit, turnover intention, and Turkey in different combinations. On the basis of past research, the factors of job satisfaction among nurses and their intention to quit the existing position or occupation are influenced by individual attribute (e.g., gender, age, marital status, education qualification, and experience) and organizational factors (e.g., pay, promotion, different types of benefits, co-worker/supervisor support, and work environment).

### Factors related to nurses’ job satisfaction

Job satisfaction is defined as the emotional feelings as well as the behavioral expression for a job. The feeling is influenced by some job related factors such as pay, different types of benefits, recognition, working condition, relation with coworker and supervisors, and others (Cowin et al., 2008; Yılmazel, 2013). Similar to other professions, it is acknowledged that nurses’ job satisfaction is inversely linked with their intention to quit their profession (Applebaum et al., 2010). Researchers discovered that highly satisfied nurses do not quit the existing job (Flinkman et al., 2008). A review on nurses’ job satisfaction expressed that job satisfaction of nurses is positively associated with job stress, depression, and organizational commitment (Lu et al., 2012). In addition, they identified that job satisfaction had a moderate relationship with some determinants e.g., role ambiguity, recognition, supervisor support, and coworker cooperation. They found a weak association with nurses’ personal characteristics for example, gender, age, years of experience, education level, and dealing with strategies. They also revealed an inconsistent impact on job satisfaction for same determinants across nations. For instance, job satisfaction was strongly correlated with individual characteristics (e.g., age) among Turkey and US nurses (Çimen & Şahin, 2000; Kavanaugh, Duffy & Lilly, 2006), but no correlation was found among nurses in China (Lu, While & Louise Barriball, 2007). Van Bogaert et al. (2010) reported that job stress was positively associated with job satisfaction among nurses in Belgium though no relationship was found among nurses in USA (Packard & Motowidlo, 1987).

Studies on nurses’ job satisfaction in different countries revealed that job satisfaction is usually moderate or low in nursing profession (Akgöz et al., 2005; Asegid, Belachew & Yimam, 2014; Sabanciogullari & Dogan, 2015; Yılmazel, 2013). Sizeable research is reported on nurses’ job satisfaction and job dissatisfaction in various settings. For example, a survey on job satisfaction of 98,110 nurses from nine countries conducted by Aiken et al. (2011) reported that job satisfaction was peak in Germany (83%) followed by USA (78%), New Zealand and Canada (67%), South Korea (64%), UK (63%), China (54%), and Japan (40%). Researchers argued that the levels of nurses’ job satisfaction may vary across samples and countries. They also stated that similar factors may not affect the level of job satisfaction in different countries (Zhu et al., 2012). In Greece, a study on cardiac certified clinical nurses revealed that 38% of nurses were unhappy with their supervisors and 34% with their colleagues (Iliopoulou & While, 2010). They also reported that 34% of them were satisfied with their hospital management, and 81% nurses expressed payment/salary as a significant job satisfaction dimension. In addition, researchers explored some factors that lead to job dissatisfaction. These are low public appreciation for nursing profession or poor image of the profession, low wages, inadequate social opportunities, poor relationship with the management, lack of job security, inadequate involvement in decision-making processes, and inflexible working hours (Aiken et al., 2013; El-Jardali et al., 2009). Other developing countries such as public health services in Malaysia indicated that the major contributors include heavy workload, repetitive work, and poor working environment. Respondents identified that inconsiderate and inequitable superior/matron, lack of recognition, and conflict within and between groups were common factors of satisfaction facets (Loo & Beh, 2012).

### Nurse turnover determinants

Currently, turnover issue among the nurses is one of the prime concerns in the healthcare sector. Keeping this in mind, researchers explored some significant factors that prompt nurses’ decision retention or to quit their profession. Applebaum et al. (2010) asserted that turnover intention of nurses is positively associated with nursing workload, stress for work, and burnout. Similarly, Meeusen et al. (2011) stated that emotional and psychological fitness of nurses deteriorated because of excessive workload and lack of coworker and supervisor support. Consequently, turnover intention is increased. A survey on job satisfaction of healthcare professionals and their intention to quit the job reported that length of professional experience is an important factor for nurses’ job satisfaction and their intention to quit from the profession (Kavanaugh, Duffy & Lilly, 2006). In addition, they found that individual characteristics (e.g., gender, age educational level, and race) are not liable for variances in job satisfaction. Recent studies indicate that nurse turnover or their intention to quit is associated with transformational leadership style (Raup, 2008) and participative governance in hospital (Gormley, 2011). Furthermore, Leiter, Price & Spence Laschinger (2010) and Delobelle et al. (2011) described that nurses are inspired for their turnover intention more by managers/supervisors than by coworkers.

A myriad of research shows that demographic characteristics are allied with nurses’ turnover intention. In recent works, researchers discovered an inverse relationship between turnover intention and individual’s age in nursing profession (Chan et al., 2009; Ma et al., 2009). It is observed that nurses in Ireland reported a greater tendency of premature retirement because of being female, kinship responsibilities, high workload, and lack of training opportunities in the workplace (McCarthy, Tyrrell & Lehane, 2007). Moreover, Delobelle et al. (2011) found that the turnover intention of nurses is inversely related with years of nursing experience in South Africa while in Jordan, job satisfaction of nurses is positively correlated with years of nursing experience and their age (Mrayyan, 2005). Similarly, Chan et al. (2009) reported that fresh graduates are highly interested to quit the current position within their first year of nursing practice. However, Beecroft, Dorey & Wenten (2008) argued that fresh graduate nurses feel committed to the organization, and turnover intention is lower given that they are happier with their professions and pay. Some recent works show that turnover intention of nurses is positively associated with higher levels of nursing education (Delobelle et al., 2011; Stewart et al., 2011). Besides, Chan et al. (2009) revealed that educational level of nurses is strongly correlated with their turnover intention or intention to stay in the organization. They reported that the higher level of education, the higher level of turnover in current position. On the contrary, Borkowski et al. (2007) stated that greater professional commitment in nursing is correlated with higher education of nursing (e.g., bachelor/master degree/PhD.). They also expressed that a highly educated nurse bears lower intention to quit the profession.

In a contemporary study, Meeusen et al. (2011) reported that lack of fairness, politics, threats to personal safety, and the risk of possible layoffs are causes of increased job dissatisfaction among nurses. In a recent study, researchers explored that job satisfaction has a moderate relationship with supervisor support, coworker support and recognition in nursing (Lu et al., 2012). Also, they reported that pay, fringe benefits, and contingent rewards are associated with nurses’ job satisfaction, and these factors are highly related with turnover intention. Some researches show that pay and financial benefits are considered as the most significant factors for job satisfaction among male nurses compared to that of female nurses (Borkowski et al., 2007; Chan et al., 2009). In addition, Leiter, Price & Spence Laschinger (2010) stated that intention to quit the nursing profession is associated with the support of nursing staff and manager’s ability. In the same way, Tourangeau & Cranley (2006) concluded that nurses are more likely to continue work in current position who perceived their team members as cohesive and supportive.

### Nursing in Turkey

Past studies have identified generally nurses’ job satisfaction is at a moderate level in Turkey (Akgöz et al., 2005; Sabanciogullari & Dogan, 2015; Yılmazel, 2013). However, these findings contradict that of Cimete, Gencalp & Keskin (2003) and Erdem et al. (2008) who found that the nurses’ job satisfaction to be the lowest among the health professional groups. A recent study indicates that the main reasons of nurses’ turnover intention is that of poor working conditions and adverse perception of nursing profession in Turkish society (Gök & Kocaman, 2011). Similarly, a survey of 397 Turkish nurses at health application and research center of Uludag university found low level of job satisfaction (Akgöz et al., 2005). In that study, the majority of nurses (68%) perceived a low job satisfaction level. Moreover, Sabanciogullari & Dogan (2015) conducted a research on 2,122 nurses in Turkey. They explored a strong positive correlation between nursing professional identity and nurses’ job satisfaction. In addition, collectively 15.5% of the nurses informed that they have intention to quit their job because of professional identity crisis and lower job satisfaction.

A recent work on 195 medical secretaries and 677 nurses conducted by Top & Gider (2013) assessed job satisfaction in Turkey. They revealed that organizational commitment has a strong positive correlation with nurses’ job satisfaction. They also reported that demographic characteristics of nurses for example gender, age, level of education, marital status, salary, years of experience in the hospital, and type of hospital ownership influence the job satisfaction of nurses. Similarly, Çimen & Şahin (2000) stated that the job satisfaction level of nurses increased in parallel with an increase in age. In addition, Aslan & Akbayrak (2002) reported that the more is the nursing experience the more is the satisfaction in nursing profession. Paşaoğlu & Tonus (2014) identified nurses’ job satisfaction at low level during the first 5-years. They also stated that nurses’ job satisfaction usually increase along with their increased working experience in hospitals. Furthermore, Yıldız, Ayhan & Erdoğmuş (2009) identified a positive effect of socio-demographic characteristics, level of job satisfaction, and motivation on nurses’ turnover intention in Turkey. They also found supervisor support as an important determinant among the organizational factors of nurses’ turnover intention. Besides, they revealed working hours as a significant factor of nurses’ intention to quit the existing job in Turkey. In this study, the job satisfaction facets were scrutinized specifically for nursing profession. The impact of socio-demographic characteristics on job satisfaction was also examined along with the intention to quit among nurses at the healthcare sector in Turkey.

## Objectives of the Study

Evaluating nurses’ job satisfaction at private hospitals of Turkey and scrutinizing their intention to quit the existing employment are the prime objectives of this study. Besides, there are some specific objectives:

∙ To identify the elements of job satisfaction among nurses.
∙ To measure the level of nurses’ job satisfaction.
∙ To measure the level of intention to quit among the nurses.
∙ To investigate the association between nurses’ job satisfaction and their intention to quit the existing workplace.
∙ To examine the effects of socio-demographic variables (e.g., as gender, age, marital status, education level, and nursing experience) on job satisfaction and intention to quit.

## Methods

### Sample and data collection procedures

In this study, a cross sectional survey was employed since this type of survey is suitable to describe relationships between variables. Six private hospitals of Kocaeli—a province of Turkey—were selected to conduct the survey. It was a face-to-face survey. The questionnaire contained a cover letter, a permission form, and the purpose of the research with brief description, and guidelines on how to fill up the questionnaire. The sample comprised of nurses employed in the pediatric ward, general ward, intensive care unit, and day ward settings. Non-probability sampling—purposive sampling-technique was employed. Usable questionnaire was 417. A total of 552 nurses participated in the study, representing 13.98% (417/2,982) of the total population from the six hospitals. The fitness criteria of the participants were ensured in all the hospitals. These six hospitals were selected because of their state-of-the-art medical facilities for large numbers of patients from different socio-economic levels. In this study, public hospitals were excluded because of the lengthy requirements to obtain permissions granted for data collection within the required study period.

### Instrumentation

A structured questionnaire was developed from the literature of earlier studies on nurses’ job satisfaction. The questionnaire was divided into three segments. The first segment comprised demographic characteristics such as gender, age, marital status, education level, working position/title, years of nursing experience, working schedule, etc. The second segment included 36 items that are related to job satisfaction developed by Spector, (1985). These items are acknowledged as the Job Satisfaction Survey (JSS). In this study, nine independent variables (i.e., pay promotion, contingent rewards, fringe benefits, operating procedures, work environment, coworkers, supervision, and communication) were represented by the 36 items. The nurses were requested to opine using a 6-point Likert scale (where, 1 = strongly disagree to 6 = strongly agree). Nearly one-fourth of the items were described in an affirmative manner and the rest of the items were in a negative manner. Hence, the scores of items in negative manner were inverted before analysis. The last segment contained 1 item. Nurses were asked to rate a 4-point Likert scale (Where, 1= very unlikely to 4 = very likely) to express their intention to quit the present workplace in the coming year.

### Reliability and acceptability estimates

Cronbach’s coefficient (*α*) for each selected facet of the JSS ranged from 0.73 to 0.92 (normally 0.6 and above is acceptable), which specifies the internal consistency of data (Spector, 1985). Firstly, a pilot test was conducted to validate the questionnaire with 30 nurses who were not incorporated in the sample. The instrument is acceptable as response rate was appreciable. Acceptability was considered in terms of missing responses rates and refusal rates (Fitzpatrick et al., 1988). Finally, the modified questionnaire was served to 650 nurses in six Turkish private hospitals. 552 nurses filled up the questionnaires and response rate was 84.92%. The response rate of each hospital varied from 90% to 98%. After analyzing the missing data, we found that 87% respondents have no missing values for the entire set of 54 items.

### Ethical considerations

Ethical approval was provided by Marmara University ethics committee (reference: MUEC/06/QQ09/07/2013), and all study works were accomplished and compliant with the national ethics regulations of Turkey. The questionnaire completion was deliberate and anonymous. The privacy of data was assured and maintained. All participants provided their written consent to participate in this study.

### Data analysis

For data analysis, SPSS (Statistical Package for the Social Sciences; version 22; Chicago, IL, USA) was used as statistical software. In this study, both descriptive and inferential statistical analyses such as regression analyses, correlation analyses, ANOVA, and post-hoc tests were performed to achieve objectives. For statistical significance, a level of *p* < 0.05 was used as a cut-off. Spearman’s rank correlation coefficient (*r*_*s*_) is calculated for correlation analyses among the variables. Lastly, Binomial logistic regression was used with the help of back-ward stepwise analysis, dichotomizing turnover intent by recoding response options (‘Likely/Very Likely’ = 1; ‘Unlikely/Very Unlikely’ = 0), as proposed by Hosmer Jr, Lemeshow & Sturdivant (2013). The predictor variables for job satisfaction and intention to quit were selected using the Wald test (correlation *p* < 0.25) along with the theoretical relevance.

## Results

A total of 417 questionnaires were completed and returned by the respondents which represent 87% of targeted nurses (total 552). Among them, male respondents totalled 155, and the remaining 262 were female nurses working at six private hospitals. The age range among the nurses who participated was 21–60 years (mean: 31.58 and standard deviation: 6.0). The average experience of these nurses was 12.7 years (standard deviation: 6.3) with a range from zero to 36 years. The descriptive statistics of these nurses are shown in Table 1.

**Table 1:** Demographic characteristics of the nurses (*n* = 417).

Particular	Percentage (%)	Frequency
*Gender*		
Male	37	155
Female	63	262
*Age group*		
21–25	23.5	98
26–30	30.7	128
31–35	19.6	82
36–40	14.0	58
41–45	7.6	32
≥46	4.6	19
*Marital status*		
Single	32.8	137
Married	55.4	231
Widowed	7.2	30
Divorced	4.6	19
*Education level*		
Diploma/Associate degree	58	242
Graduate (Baccalaureate)	35.3	147
Master of science	6.7	28
*Working experience*		
≥5 year	23.5	98
6–15year	32.6	136
16–25 year	26.9	112
26–35 years	10.1	42
≤36 years	6.9	29
*Unit*		
General Ward	25.7	107
Pediatric Ward	7.2	30
Incentive care	24.2	101
Day Ward	42.9	179
*Schedule*		
Permanent morning	24.2	101
Permanent night	18.5	77
Rotating day	57.3	239

The JSS items along with their subscales (facets) were listed in Table 2 in an ascending order. In order to focus on their magnitude to respondents’ job satisfaction, mean and standard deviation for each item was also presented accordingly. If the mean value for any item is more than or equal to four, this means that the respondents are satisfied for that particular item. Again, if an item scores less or equal to 3, it appears that the respondents were dissatisfied with that particular item. A mean value in between 3 and 4 show ambivalence; thereby, the calculation is the percentage of mean value divided by the mean of maximum possible total (that is 6) which specifies respondents’ level of satisfaction for that item. Table 2 also incorporates the percentage of nurses who gave responses of “moderately agree” or “strongly agree,” with the purpose of determining the factors that are allied with the highest level of satisfaction.

**Table 2:** The means and standard deviations for all items of nine facets of the job satisfaction survey.

Subscale	Items	Mean	Standard deviation	Rating nurses with opinion as strongly agree or moderately agree (%)	Satisfaction ranking (descending)	Facet Mean of satisfaction	Facet standard deviation
Pay	Pay1	2.79	1.60	18.7	30	3.25	0.16
	Pay2	3.26	1.53	16.2	34		
	Pay3	3.28	1.31	23.0	23		
	Pay4	3.45	1.61	31.9	11		
Promotion	Pro1	3.01	1.35	18.7	31	3.34	0.25
	Pro2	3.30	1.30	26.3	15		
	Pro3	3.52	1.64	23.7	20		
	Pro4	3.54	1.48	28.0	13		
Supervision	Sup1	3.79	1.58	40.0	4	3.87	0.15
	Sup2	3.79	1.47	34.4	9		
	Sup3	3.81	1.52	35.0	7		
	Sup4	4.17	1.53	43.2	3		
Fringe benefits	Fri1	2.64	1.23	12.4	35	3.20	0.28
	Fri2	3.20	1.40	21.9	24		
	Fri3	3.21	1.51	19.0	28		
	Fri4	3.53	1.36	23.7	21		
Contingent rewards	Con1	3.01	1.47	18.7	33	3.14	0.12
	Con2	3.08	1.44	18.7	32		
	Con3	3.19	1.45	21.9	26		
	Con4	3.29	1.38	21.9	25		
Operating conditions	Ope1	2.69	1.24	11.2	36	3.25	0.46
	Ope2	3.34	1.46	24.0	18		
	Ope3	3.44	1.58	25.6	17		
	Ope4	3.64	1.39	24.4	19		
Coworkers	Cow1	3.28	1.44	26.3	16	3.84	0.59
	Cow2	3.39	1.33	21.2	27		
	Cow3	4.27	1.39	43.9	2		
	Cow4	4.21	1.67	52.7	1		
Work environment	Nat1	3.67	1.46	30.7	12	3.89	0.15
	Nat2	3.90	1.63	34.4	10		
	Nat3	3.95	1.43	35.7	6		
	Nat4	4.23	1.47	38.8	5		
Communication	Com1	3.13	1.65	23.7	22	3.35	0.31
	Com2	3.13	1.42	19.3	29		
	Com3	3.33	1.33	27.5	14		
	Com4	3.79	1.59	35.0	8		

From Table 2, it is evidenced that the mean satisfaction level for the respondents was 3.46 out of 6 (standard deviation = 0.42), which means that the average satisfaction level is 58%. From the table, it is also evident that higher scores were assigned by respondents to specific items such as “I like my work environment,” “my coworkers are comfortable with me,” and “I enjoy the administration of my supervisor”. However, lower scores were assigned to the items i.e., “I feel my efforts are not rewarded properly,” “I feel heavy workload pressure,” and “I am not satisfied with my salary.” According to the results from Table 2, the highest satisfactory facets were “work environment” “supervisor support,” and “coworkers,” while “fringe benefits” and “contingent rewards” were the least satisfactory facets among nurses.

We also tested nurses’ intention to quit the existing job settings with relation to present job satisfaction by asking “Considering your career aims, do you want to change your present workplace in the coming year?” The results for this single statement are shown in Table 3. It is shown that the mean intention to quit score was 2.81 out of 4 (standard deviation = 0.62), which means that 60.9% nurses reported that they want to quit the present workplace within one year. So, in Table 3, it is observed that 24.4% nurses were reported that they “very likely” to quit their present workplace in the next year. Similarly, 36.5% respondents reported that they are “likely” to quit their present job settings in the next year. Moreover, it is revealed that job satisfaction of nurses was strongly and negatively associated with turnover decision (*r* = − 0.723, *p* < 0.01).

**Table 3:** Descriptive values for nurses’ intent to quit the present workplace.

Level	Frequency	Percentage (%)
Very unlikely	93	22.3
Unlikely	70	16.8
Likely	152	36.5
Very likely	102	24.4

Scheffé’s method was run for examining the relationship between job satisfaction of nurses on their promotion and work unit. It was found that Scheffé’s *F* score 6.71 (*p* = 0.03) with the correlation *r* = 0.22 (*p* = 0.01). While testing post-hoc for the possible interaction (family alpha = 0.05), the results revealed that general ward nurses’ satisfaction level was higher than that of pediatric ward, intensive care unit, and day ward. This result was significant, since *p* = 0.045. The correlation between nurses’ fringe benefit and work unit was also found significant (*r* = − 0.25, *p* = 0.001) and the Scheffé’s F score was 11.68 with *p* = 0.03. While testing post-hoc for the possible interaction (family alpha = 0.05), the results revealed that day ward nurses’ fringe benefits were higher than that of pediatric ward, general ward, and intensive care unit (*r* = − 0.18, *p* = 0.001). Now, Scheffé’s *F* score for contingent rewards and work unit was 10.59 (*p* = 0.001) with correlation *r* = − 0.19 (*p* = 0.001). The mean satisfaction for contingent rewards was found higher among the general ward nurses, than that of pediatric ward, intensive care, and day ward nurses (*p* = 0.001). This was tested for the post-hoc testing of the possible interactions at a family alpha of 0.05.

The differences between the two main study variables (job satisfaction and intention to quit of nurses) with relations to major demographic variables were also examined. First, independent *t*-test was run to examine whether there is any differences based on “gender” of the nurses. The results revealed that male participants ((mean = 3.44, standard deviation =0.52), *t* = 0.63, *p* = 0.002) were scored slightly, but not significantly lower than the female respondents (mean = 3.48, standard deviation =0.31) in terms of their present job satisfaction level. However, an opposite result was found in case of nurses’ intention to quit the present workplace, where female participants ((mean =2.98, standard deviation = 0.62), *t* = 0.54, *p* = 0.03) were scored higher than their counterpart (mean = 2.64, standard deviation =0.51).

Next, the difference in job satisfaction and intention to quit was determined with respondents’ marital status. The results from sample *t*-test revealed a significant difference in job satisfaction; married participants ((mean = 3.82, standard deviation = .41), *t* = − 2.21, *p* = 0.000) were scored significantly higher than single participants (mean = 3.10, standard deviation = 0.32). Moreover, a significant difference in intention to quit based on respondents’ marital status; single participants ((mean = 2.90, standard deviation = 0.63), *t* = 0.72, *P* = 0.003) were scored moderately higher than married participants (mean = 2.72, standard deviation = 0.52).

In order to test the differences in job satisfaction and intention to quit based on different educational backgrounds of nurses, we used Scheffé’s method. It is observed that nurses’ job satisfaction and educational level (*r* = − 0.35, *p* < 0.001) with Scheffé’s *F* score 11.58 (*p* < 0.001). In post-hoc testing of the possible interactions at a family alpha of 0.05, the mean satisfaction of nurses with diploma degree was higher than nurses with bachelor degree and master degree holders (*p* < 0.001). On the other hand, it is observed that nurses’ intention to quit and their educational level (*r* = 0.26, *p* = 0.01) with Scheffé’s *F* score 5.76 (*p* < 0.001). In post-hoc testing of the possible interactions at a family alpha of 0.05, the mean intention to quit with master degree holder was higher than nurses with diploma degree, and bachelor degree holders (*p* < 0.001).

Additionally, Table 4 summarizes the correlation between variables. The Spearman’s rank correlation coefficient (*r*_*s*_) was tested to examine the relationships between job satisfaction and nine organizational variables, or five demographic variables. A similar test was run with intention to quit and same variables. Job satisfaction was significantly and positively correlated with age and experience. Moreover, no association was found between job satisfaction and gender, marital status or education level. Intention to quit was significantly and negatively correlated with age and experience; the older and more experienced nurses reported less intention to quit than younger nurses, and significantly positively associated with education level of nurses.

**Table 4:** Spearman’s rank correlation.

	Variable	1	2	3	4	5	6	7	8	9	10	11	12	13	14	15	16
1	Gender	1.00															
2	Age	0.19	1.00														
3	Marital status	0.25	0.32	1.00													
4	Education level	0.21	0.27	−0.35	1.00												
5	Experience	0.16	0.28^*^	0.31	0.27	1.00											
6	Pay	0.31	0.27	0.23	0.37	0.28	1.00										
7	Promotion	0.23	−0.37	0.24	−0.17^**^	0.27	0.17	1.00									
8	Supervisory support	0.24	−0.17	0.14^*^	0.18	−0.37	−0.16	0.31	1.00								
9	Fringe benefits	0.14	0.18^**^	−0.17	0.15	−0.17^*^	0.24	0.28	−0.15	1.00							
10	Contingent rewards	−0.17	0.15	0.14^*^	0.23	0.14	0.31	0.27^*^	−0.14^*^	0.17	1.00						
11	Operating conditions	−0.15	0.27	0.28	0.24	−0.17	0.23	0.24	−0.17	0.23^*^	0.31	1.00					
12	Coworkers	−0.26	−0.18	0.27	0.14	−0.15	0.24	0.14^**^	−0.15	0.24	0.23^*^	0.18	1.00				
13	Work environment	−0.27	−0.17	−0.37	−0.17	−0.24	0.28	−0.17	−0.24	0.28	0.24	0.15	0.12	1.00			
14	Communication	0.18	0.25	−0.17	−0.15	−0.18	0.27	0.14	0.23	0.24	−0.17	0.23	0.31	0.15	1.00		
15	Job satisfaction	0.60	0.31^**^	0.28	0.25	0.66^**^	0.87^***^	0.67^***^	0.64^**^	0.84^***^	0.55^***^	0.74^***^	0.53^***^	−0.75^**^	0.59^**^	1.00	
16	Intention to quit	0.18	−0.37^***^	0.17	0.57^***^	−0.45^**^	−0.17	−0.26	0.14	−0.17	−0.24	0.28	0.24	−0.21	0.19	−0.57^**^	1.00

**Notes.**

* *P* < 0.05 level (two-tailed).

** *P* < 0.01 level (two-tailed).

*** *P* < 0.001 level (two-tailed).

Table 5 presents the results of stepwise backward logistic regression of intention to turnover as shown by Hosmer Jr, Lemeshow & Sturdivant (2013). Backward logistic regression of intention to quit on job satisfaction, controlling for the effect of gender, age, marital status, education, and experience resulted in a significant model (*x*^2^ = 25.78, *d*.*f*. = 3, *P* < 0.001) with a 64.47% correct classification rate of predicted values. Job satisfaction, age and education level were found to predict turnover intent significantly, suggesting that younger and higher educated nurses with less job satisfaction were more likely to consider turnover. Higher educated nurses were more than twice as likely to consider turnover, and every one unit increase on the job satisfaction scale was associated with being 61% less likely to consider turnover as shown in Model 1 of Table 5.

**Table 5:** Stepwise backward logistic regression of intention to quit.

	*B*	SE	Wald (*d*.*f*. = 1)	*P*	OR (95%CI)
Model 1^a^					
Age	−0.07	0.01	11.01	0.001	0.92 (0.74–0.85)
Education level	0.95	0.47	4.62	0.029	2.47 (1.12–5.78)
Job satisfaction	−0.91	0.37	5.91	0.001	0.33 (1.24–0.65)
Constant	5.07	1.41	8.82	0.002	
−2 log likelihood	142.08				
Correct classification rate (%)	64.47				
Model 2^b^					
Age	−0.07	0.01	11.24	0.001	0.92 (0.74–0.85)
Education level	0.87	0.34	5.24	0.011	2.34 (1.05–0.95)
Supervisory support	−0.56	0.17	4.89	0.001	0.71 (0.54–0.87)
Work environment	−0.54	0.25	6.34	0.034	0.82 (0.42–0.97)
Coworkers	−0.72	0.36	4.42	0.042	0.43 (0.14–2.65)
Constant	6.95	2.07	8.99	0.008	
−2 log likelihood	135.85				
Correct classification rate (%)	66.12				

**Notes.**

a Variables entered: gender, age, marital status, education level, experience (step 1); job satisfaction (step 2).

b Variables entered: gender, age, marital status, education level, experience (step 1); satisfaction, pay, promotion, supervisory support, fringe benefits, contingent rewards, operating conditions, coworkers, work environment, communication (step 2).

When substituting the composite measure of job satisfaction by its facets, as indicated in Model 2 of Table 5, supervisor support was the only facet significantly predicting turnover intent, next to age and higher education. Nurses who reported more satisfaction with supervisor support were nearly 39% less likely to consider a job change. Satisfaction with work environment, supervisor support and coworkers was also retained, but without statistical significance. The model was highly significant (*x*^2^ = 28.78, *d*.*f*. = 4, *P* < 0.001) with a correct classification rate of 66.12%.

In summary, a statistically-significant negative relationship is revealed from the results between the two main study variables job satisfaction and intention to quit among the nurses. A significant positive rapport is found between nurses’ job satisfaction and age. Similar results are also reported for nursing experience. But a significant negative relationship with intention to quit is found for age and nursing experience. Female nurses and married nurses have higher job satisfaction than unmarried nurses, and male nurses. For intention to quit, male nurses and unmarried nurses were found to have higher score than female nurses and married nurses. Nurses with diploma were found to have the highest level of job satisfaction in comparison to others and the nurses with master degree were found to be the lowest. While considering intention to quit, the nurses with master degree were found to have the highest level possibilities, while the nurses with bachelor degree were scored the lowest. Moreover, intention to quit was statistically significantly explained by job satisfaction, age and education (*P* < 0.001), with younger and higher educated nurses being more likely to show turnover intent. Satisfaction with work environment was the only facet significantly explaining turnover intent when controlling for gender, age, marital status, education, and experience (*P* < 0.001).

## Discussion

This study filled a significant gap in the existing knowledge of nurses’ job satisfaction and their intention to quit in Turkey. This study found that nurses’ job satisfaction score was 3.46 (out of 6) i.e., the average satisfaction level was 58%. Therefore, the job satisfaction of Turkish nurses was at moderate level. This result is consistent with earlier studies on Turkish nurses (Akgöz et al., 2005; Sabanciogullari & Dogan, 2015; Yılmazel, 2013). However, this result contrasted with the study of Erdem et al. (2008), who revealed that most nurses in Turkey were dissatisfied with their profession. In addition, the present study concluded that job satisfaction among nurses is significantly and negatively associated with intention to quit. The finding is similar to the survey of Turkey nurses (Gök & Kocaman, 2011), nurses working in American settings (Applebaum et al., 2010), study of Greek nurses (Iliopoulou & While, 2010), and survey study of English nurses (Frijters, Shields & Price, 2007). In addition, 60.9% of nurses reported their strong intention to quit their existing job place in the next year.

Among the nine facets of job satisfaction, nurses collectively expressed high level of satisfaction with work environment, supervisor support, and coworkers. However, contingent rewards, fringe benefits, and pay were reported with low satisfaction; these factors were highly related to their intention to quit. This study is also congruent with Iliopoulou & While (2010) who identified supervisor support as an important factor of nurses’ job satisfaction. Moreover, they stated lower pay and financial benefits as causes of dissatisfaction among nurses consistent with this study. In addition, the recent study supported that nurses’ intention to quit the existing workplace is influenced more by managers or supervisors than by coworkers (Delobelle et al., 2011). Likewise, studies on general nurses conducted by Chan et al. (2009) and Heinen et al. (2013) found high satisfaction with coworkers but dissatisfaction with their extrinsic rewards and professional opportunities. Moreover, Leiter, Price & Spence Laschinger (2010) stated that nurses’ intentions to continue the current employment was related to manager’s capability and care of nursing staff. The results of present study supports the finding of Tourangeau & Cranley (2006) who identified that nurses felt more satisfaction and more likely to stay in hospitals when they perceived their coworkers as cohesive and supportive. In the contemporary research on Turkey conducted by Yıldız, Ayhan & Erdoğmuş (2009), supervisor support is noted as the third major impact on the nurses’ intention to quit.

The recent studies on nurses’ job satisfaction indicate that pay, promotion, contingent rewards, and fringe benefits play a significant role in job satisfaction (Han & Jekel, 2011; Top & Gider, 2013) that are consistent with the present findings. In the present study, nurses of general ward reported a higher satisfaction level in terms of promotion and contingent rewards than nurses who work in other units, and nurses of day wards reported higher satisfaction in term of fringe benefits than nurses who work in other wards. Generally, most nurses were not satisfied with their terms of promotion and fringe benefits. Interestingly, Seo, Ko & Price (2004) reported that Korean nurses would become very unhappy if they perceived that nurses in the same position elsewhere were being better rewarded. In the same way, Frijters, Shields & Price (2007) revealed that Chinese nurses were displeased with pay and promotions. In contrast, Chan et al. (2009) revealed an insignificant relation between pay and nurses’ job satisfaction. They also stated that nurses were most displeased because of unfair promotion policy, lower career advancement opportunities, and less chances of liberated work that highlights decision making, critical thinking, autonomy, and delegation proficiency aspects of nursing.

It was discovered that nurses’ job satisfaction and their intention to quit the present workplace are significantly influenced by personal characteristics of nurses. In this study, nurses reported that job satisfaction was positively associated with age. The results are congruent with the past researches of Çimen & Şahin (2000) who indicates the job satisfaction level of Turkish nurses increases in parallel with their increasing age, while Chinese nurses reported a very weak relationship between job satisfaction and age (Lu, While & Louise Barriball, 2007). Usually, one’s expectations become more realistic along with his/her maturity. The present study found a significant and negative correlation between nurses’ intention to quit and nurses’ age, which is consistent with recent research (Delobelle et al., 2011; Ma et al., 2009). Furthermore, female and married nurses reported higher job satisfaction, while male and single nurses expressed higher intention to quit the current workplace. These findings are congruent with prior research (Top & Gider, 2013; Torkelson & Seed, 2011). In terms of intention to quit, it was shown that financial benefits are imperative factors of intention to quit for males nurses compared to females (Borkowski et al., 2007; Kacel, Miller & Norris, 2005) that supports the present findings. Similarly, Lu et al. (2012) identified single male nurses are more exposed to burnout than married nurses.

Nurses reported that professional experience is positively correlated to their job satisfaction i.e., nurses with less work experience are more dissatisfied with their job compared with experienced nurses, which is congruent with the earlier research of Kavanaugh, Duffy & Lilly (2006). Past researchers claimed that years of nursing experience and age are related variables for one’s job satisfaction that is also consistent with the present results. Usually, younger nurses who have not as much of professional experience are more displeased with their workload, pay, financial benefits, promotion, professional support, and the opportunity to continue their education (Torkelson & Seed, 2011). It is reported that the experienced nurses (more than 15 years) conveyed less intention to quit from the existing workplace compared with younger nurses. Similarly, some contemporary studies stated that young nurses especially fresh graduates have higher intention to quit the existing workplace within the first year of practice (Beecroft, Dorey & Wenten, 2008; Chan et al., 2009; Delobelle et al., 2011). However, the present findings are not similar to earlier results of Mrayyan (2005). Also, Lu et al. (2012) stated that the intention to quit of nurses is weakly associated with year of age and years of job experience.

Chan et al. (2009) revealed that nurses’ educational levels are not strongly related to job satisfaction and intention to quit. The present study reveals an opposite result. Nurses with master degree were the most dissatisfied nurses with their job than nurses with diploma and bachelor degrees. In addition, nurses with a master’s degree have a higher level of intention to quit the current employment than others. Similarly, some prior research explored that highly educated nurses are more likely to quit the existing workplace. They are conscious of their career advancement, and seek alternative employment opportunities because of inadequate work opportunities and benefits in their current organization (Delobelle et al., 2011; Stewart et al., 2011). However, the present study rejects the finding of Borkowski et al. (2007) who stated that higher education level (for example, master degree in nursing) is correlated with enhanced professional commitment and possibility of intention to quit the existing employment as well as the nursing profession. In the same way, Liu et al. (2012) did not find any strong relation between nurses’ job satisfaction and their educational qualification.

The job satisfaction factors of Turkish nurses were examined carefully to enhance their satisfaction in organizations. Consequently, the likelihood of intention to quit among nurses will be reduced. The present findings are consistent with previous researches conducted in other nations. The significant difference among the nations seems to be the ranking of certain job satisfaction variables over others and cultural dissimilarities which is innate in the healthcare delivery systems of different nations.

## Implications for the Nurse Managers

Implementation of proper motivation programs would enhance job satisfaction and decrease intention of Turkish nurses to quit, which ultimately may increase healthcare service quality. Nursing managers can practice co-management models for nurses. Nurses can take part in professional activities such as contribution in decision making and sense of confidence in nursing practices to increase their sense of belonging in the organization (Aiken et al., 2013; Flinkman, Leino-Kilpi & Salanterä, 2010). Consequently, their job satisfaction can be increased. Evidently, it is important that nursing administrators and employers should inspire and allocate adequate resources for professional development of nurses, thus they can avail and take part in constructive programs and meetings (Aiken et al., 2013; Asegid, Belachew & Yimam, 2014).

Retaining the qualified nurses is a challenge in all organizations. To retain nurses, managers should take initiatives to identify and assess the job satisfaction factors of nurses. Managers ought to routinely screen for indications of dissatisfaction by conducting job satisfaction surveys. Sometimes, nurses’ intent to apply positions on different units or hospitals may be one of the first signs of their job dissatisfaction. To increase job satisfaction and retention, managers ought to ensure professional opportunities such as working with skilled peers, providing unceasingly support to nurses, endorsing collaborative nurse–physician relationships, securing adequate staffing, advocating and helping control over nursing practice, increasing clinical autonomy, and promoting nurse education (Hayes, Bonner & Pryor, 2010).

Highlighting the factors of nurses’ job satisfaction, the present study suggests a suitable and strategic plan of professional development for Turkish nurses so that the probability of intention to quit may be minimized from their existing workplace as well as the nursing profession. The present study provides a guideline for hospital management to identify the positive and negative factors of job satisfaction among nurses. By identifying the positive factors of job satisfaction, management will ensure its continuance. Addressing negative factors among healthcare policy makers and hospital management are necessary and crucial measures to improve nurses’ job satisfaction resulting in the reduction of nurses’ intention to quit.

## Conclusion

Job satisfaction is a noteworthy issue for ensuring proper nursing care in healthcare sector. Furthermore, job satisfactions of nurses will ensure better quality healthcare services and enhance their professional commitment. The present study explored the important factors of job satisfaction among the nurses who were employed in private hospitals in Turkey. Nurses collectively expressed their job satisfaction at a moderate level. The findings of this study demonstrated the significance of fair promotion opportunity, fringe benefits, and contingent rewards as important factors in improving job satisfaction and retention among nurses. Amongst the nine job satisfaction factors, nurses reported a higher satisfaction level with their work environment, supervisors, and coworkers. This indicates that the work environment, responsive and cooperation among co-workers as important factors to the nurses’ job satisfaction. Job satisfaction and retention among nurses varies accordingly with demographic variables. The findings of this study are comparable with other studies in different nations. According to earlier research, job satisfaction is negatively interrelated with intention to quit among nurses which is congruent with the contemporary studies. Thus, low satisfaction hampers the quality of healthcare services and builds the intention to quit the organizations. Consequently, organizations might suffer from different direct and indirect factors of satisfaction.

## Limitations and Future Research

In this study, the sample size was relatively small and limited to nurses who were employed among the private hospitals in the province of Kocaeli, Turkey. Thus the findings are applicable to this sample only. For future research, a larger and representative random sample of nurses from both private and public hospitals can be investigated. Although the findings of this study are congruent with past studies, caution is required in drawing any firm conclusions for other nations. Further examination is encouraged owing to the multidimensional nature of both concepts—job satisfaction and intention to quit. An ethnographic qualitative research approach would be better addressed in which nurses can express their experiences and needs. This approach may permit the academics to achieve a comprehensive understanding of both cultural and individual nurses’ point of views.

## Supplemental Information

10.7717/peerj.1896/supp-1 Data S1 Raw dataset

## Additional Information and Declarations

### Data Availability

The following information was supplied regarding data availability: The raw data has been supplied as Data S1.
