# Supplementary material for: Job satisfaction and intention to quit: an empirical analysis of nurses in Turkey
Source: PeerJ. 2016 Apr 26;4:e1896. doi: 10.7717/peerj.1896 (PMC4860322; doi:10.7717/peerj.1896)
Supplement: Data S1 [file peerj-04-1896-s001.pdf]

Supporting Information

<u>Demographic results</u>

Particular	Percentage (%)	Frequency
Gender		
Male	37	155
Female	63	262
Age Group		
21-25	23.5	98
26-30	30.7	128
31-35	19.6	82
36-40	14.0	58
41-45	7.6	32
≥ 46	4.6	19
Marital status		
Single	32.8	137
Married	55.4	231
Widowed	7.2	30
Divorced	4.6	19
Education level		
Diploma/ Associate degree	58	242
Graduate (Baccalaureate)	35.3	147
Master of science	6.7	28
Working experience		
≥ 5 year	23.5	98
6 – 15 year	32.6	136
16 – 25 year	26.9	112
26 – 35 years	10.1	42
≤ 36 years	6.9	29
Unit		
General Ward	25.7	107
Pediatric Ward	7.2	30
Incentive care	24.2	101
Day Ward	42.9	179
Schedule		
Permanent morning	24.2	101
Permanent night	18.5	77
Rotating day	57.3	239

Supporting Information

Response for job satisfaction factors

Response to questionnaire (1-12)

	Pay1	Pay2	Pay3	Pay4	Pro1	Pro2	Pro3	Pro4	Sup1	Sup2	Sup3	Sup4
Respondent 1	2	2	1	2	1	3	3	3	4	2	2	4
Respondent 2	3	3	2	3	2	3	5	4	3	4	4	3
Respondent 3	2	2	3	2	2	4	1	2	3	1	3	5
Respondent 4	2	2	4	2	2	3	3	2	3	2	5	5
Respondent 5	3	3	1	3	3	5	4	4	4	4	5	4
Respondent 6	1	3	2	3	1	1	2	1	3	3	4	5
Respondent 7	2	4	2	4	2	3	4	3	5	4	3	6
Respondent 8	1	3	2	3	2	4	2	4	2	2	5	4
Respondent 9	2	5	3	4	2	3	4	2	3	2	6	3
Respondent 10	2	1	4	1	1	3	3	2	4	4	4	5
Respondent 11	3	3	1	3	2	2	5	3	2	1	3	5
Respondent 12	3	4	2	4	2	3	1	4	4	3	5	6
Respondent 13	3	1	3	2	2	3	3	1	2	4	2	3
Respondent 14	1	2	2	2	3	4	4	3	4	2	4	5
Respondent 15	1	2	2	4	4	3	2	4	3	2	3	5
Respondent 16	1	2	3	1	1	5	3	2	5	4	5	4
Respondent 17	2	3	3	3	2	1	1	2	4	1	5	5
Respondent 18	3	4	4	4	3	3	3	2	3	3	4	6
Respondent 19	4	1	3	2	2	4	5	3	3	4	1	4
Respondent 20	1	2	4	2	2	2	3	4	3	1	2	3
Respondent 21	1	3	1	4	1	3	4	1	4	3	6	5
Respondent 22	5	2	3	1	1	3	2	3	4	2	4	5
Respondent 23	1	2	4	3	2	4	2	3	3	4	3	6
Respondent 24	2	3	2	4	2	2	4	4	5	1	5	3
Respondent 25	1	3	2	1	2	3	1	2	2	2	3	5
Respondent 26	2	4	1	3	3	3	3	2	3	4	4	5
Respondent 27	3	3	2	4	1	4	4	4	4	3	2	4
Respondent 28	2	4	3	2	2	3	2	1	2	3	3	5
Respondent 29	2	1	4	2	2	5	2	3	4	3	5	6
Respondent 30	3	3	1	4	2	1	4	4	2	4	2	4
Respondent 31	1	4	2	1	1	3	1	2	4	3	3	4
Respondent 32	2	2	3	3	2	4	3	2	3	5	5	3
Respondent 33	1	2	2	4	2	2	4	3	5	2	4	5
Respondent 34	2	1	2	2	2	3	1	4	4	3	3	5
Respondent 35	2	2	3	4	3	3	3	3	3	4	5	6
Respondent 36	3	3	3	1	4	4	4	4	3	2	5	3
Respondent 37	3	4	4	3	1	3	2	2	3	4	4	5
Respondent 38	3	1	3	4	2	5	2	2	4	2	3	5
Respondent 39	1	2	1	2	3	1	4	4	3	4	5	4
Respondent 40	1	3	2	2	2	3	1	1	5	3	6	3
Respondent 41	1	2	3	4	2	4	3	3	2	5	4	5
Respondent 42	2	2	4	1	1	2	4	4	3	4	3	3
Respondent 43	3	3	1	3	2	3	2	2	4	3	5	5
Respondent 44	4	3	2	4	2	1	4	2	2	3	2	5
Respondent 45	1	4	2	2	2	2	3	3	4	3	4	6
Respondent 46	1	3	2	4	3	3	4	4	2	4	3	3

Respondent 47	5	1	3	4	1	4	2	3	4	4	4	5
Respondent 48	1	2	4	2	2	1	2	4	3	3	3	5
Respondent 49	2	3	3	2	2	2	4	2	5	5	4	4
Respondent 50	1	4	2	3	2	2	1	2	4	2	1	3
Respondent 51	2	1	3	2	1	2	3	4	3	3	2	5
Respondent 52	3	2	2	2	2	1	4	1	3	3	6	5
Respondent 53	2	2	4	3	2	2	2	3	3	3	4	6
Respondent 54	2	2	1	3	2	3	2	4	4	4	3	3
Respondent 55	3	3	2	2	3	3	4	2	3	3	5	5
Respondent 56	1	4	2	3	4	3	1	2	5	5	3	5
Respondent 57	2	3	2	2	1	4	3	3	2	2	4	4
Respondent 58	1	2	3	2	2	3	4	4	3	3	2	5
Respondent 59	2	3	4	3	3	5	1	3	4	4	5	6
Respondent 60	2	2	1	3	2	1	3	4	2	2	5	4
Respondent 61	3	2	2	2	2	3	4	2	4	4	2	3
Respondent 62	3	3	3	3	1	4	2	2	2	2	3	5
Respondent 63	3	2	2	2	2	2	2	4	4	4	5	5
Respondent 64	2	3	2	2	2	3	4	1	3	3	4	6
Respondent 65	3	2	3	3	2	3	1	3	5	5	3	3
Respondent 66	2	2	3	2	3	4	3	4	2	4	5	5
Respondent 67	2	3	4	2	1	3	4	2	3	3	5	5
Respondent 68	3	3	2	3	2	5	2	2	3	3	4	4
Respondent 69	1	4	3	3	2	1	4	3	3	3	3	3
Respondent 70	2	3	2	4	2	3	3	4	4	4	5	5
Respondent 71	1	5	2	3	1	4	4	3	3	4	6	5
Respondent 72	2	1	3	4	2	2	2	4	3	3	4	6
Respondent 73	2	3	3	1	2	3	2	2	2	5	3	3
Respondent 74	3	4	4	3	2	1	4	4	3	2	5	5
Respondent 75	3	1	3	4	3	2	1	1	4	3	2	5
Respondent 76	3	2	1	2	4	3	3	3	2	3	4	4
Respondent 77	1	2	2	2	1	4	4	4	4	3	3	5
Respondent 78	1	2	3	4	3	1	2	2	2	4	5	6
Respondent 79	1	3	4	1	2	3	2	2	4	3	5	3
Respondent 80	2	4	1	3	2	3	4	3	3	5	4	5
Respondent 81	3	1	2	4	1	4	1	4	5	2	1	5
Respondent 82	4	2	2	2	2	3	3	1	4	3	2	4
Respondent 83	1	3	2	2	2	5	4	3	3	4	6	3
Respondent 84	1	2	3	4	2	1	1	4	3	2	4	5
Respondent 85	5	2	4	2	3	3	3	2	3	4	3	3
Respondent 86	1	3	2	2	3	4	4	2	4	2	5	5
Respondent 87	2	3	3	3	2	2	2	2	3	4	3	5
Respondent 88	1	4	2	3	2	3	2	3	5	3	4	6
Respondent 89	2	3	2	4	1	3	4	4	2	5	2	3
Respondent 90	3	4	3	3	2	4	1	1	3	4	4	5
Respondent 91	2	1	3	4	2	3	3	3	4	3	3	5
Respondent 92	2	3	4	2	2	5	4	3	2	3	2	4
Respondent 93	3	4	3	2	3	1	2	4	4	3	3	3
Respondent 94	1	2	1	4	3	3	4	2	2	4	5	5
Respondent 95	2	2	2	1	2	4	3	2	1	4	4	5
Respondent 96	1	1	3	3	2	2	4	4	3	3	3	6
Respondent 97	2	2	4	4	1	3	2	1	4	2	5	3
Respondent 98	2	3	1	1	2	1	2	2	2	2	5	5
Respondent 99	3	4	2	3	2	2	4	4	4	4	4	5
Respondent 100	3	1	2	4	2	3	1	1	3	1	3	4

Respondent 101	3	2	2	2	3	4	3	3	4	3	5	5
Respondent 102	1	3	3	2	3	1	4	4	2	4	6	6
Respondent 103	1	2	4	4	2	3	2	2	2	2	4	4
Respondent 104	1	2	2	1	2	3	2	2	4	2	3	3
Respondent 105	2	3	3	3	1	4	3	3	1	4	5	5
Respondent 106	3	3	2	4	2	3	4	4	3	1	2	5
Respondent 107	4	4	2	2	2	5	1	1	4	3	4	6
Respondent 108	1	3	3	4	2	1	3	3	2	4	3	3
Respondent 109	1	1	3	1	3	3	4	4	2	1	5	5
Respondent 110	5	1	4	3	3	4	2	2	4	3	5	5
Respondent 111	1	2	3	4	2	4	2	2	1	2	4	4
Respondent 112	2	3	1	2	3	2	4	2	3	4	1	3
Respondent 113	1	2	2	4	2	3	1	3	4	1	2	5
Respondent 114	2	2	3	2	2	3	3	4	1	2	6	5
Respondent 115	3	3	4	2	1	4	4	1	3	4	4	6
Respondent 116	2	3	1	4	2	3	2	3	3	3	3	3
Respondent 117	2	4	2	1	2	5	4	3	2	3	5	5
Respondent 118	3	3	2	3	2	1	3	4	3	3	3	5
Respondent 119	1	5	2	4	3	3	4	2	4	4	4	4
Respondent 120	2	1	3	1	3	4	2	2	2	3	2	5
Respondent 121	1	3	4	3	2	2	2	4	4	5	5	6
Respondent 122	2	4	2	4	2	3	4	1	2	2	5	5
Respondent 123	2	1	3	2	1	1	1	2	4	3	2	6
Respondent 124	3	2	2	2	2	2	3	4	3	4	3	3
Respondent 125	3	1	2	4	2	3	4	1	5	2	5	5
Respondent 126	3	2	3	1	2	4	2	3	4	4	4	5
Respondent 127	1	3	3	3	3	1	2	4	3	2	3	4
Respondent 128	1	4	4	4	3	3	3	2	3	4	4	3
Respondent 129	1	1	3	2	2	3	4	2	3	3	5	5
Respondent 130	2	2	1	4	2	4	1	3	4	5	4	5
Respondent 131	3	3	2	1	1	3	3	4	3	2	3	6
Respondent 132	4	2	3	3	2	5	4	1	5	2	5	5
Respondent 133	1	2	4	4	2	1	2	3	2	4	6	6
Respondent 134	1	3	1	2	2	3	2	4	3	1	4	3
Respondent 135	5	3	2	4	3	4	4	2	4	3	3	5
Respondent 136	1	4	2	2	3	2	1	2	2	4	5	5
Respondent 137	2	3	2	2	3	4	3	2	4	2	2	4
Respondent 138	1	5	3	4	2	2	4	3	2	2	4	3
Respondent 139	2	1	4	1	2	3	2	4	1	4	3	5
Respondent 140	3	3	2	3	1	3	4	1	3	1	5	5
Respondent 141	2	4	3	4	2	4	3	3	4	3	5	6
Respondent 142	2	1	2	1	2	3	4	3	2	4	4	5
Respondent 143	3	2	2	3	2	5	2	4	4	1	1	6
Respondent 144	1	2	3	4	3	1	2	2	3	3	2	3
Respondent 145	2	2	3	2	3	3	4	2	3	2	6	5
Respondent 146	1	3	4	2	2	4	1	4	2	4	4	5
Respondent 147	2	4	3	4	2	2	3	1	3	1	3	4
Respondent 148	2	1	1	1	1	3	4	2	4	2	5	5
Respondent 149	3	2	2	3	2	1	2	4	2	4	3	6
Respondent 150	3	3	3	4	2	2	2	1	4	3	4	4
Respondent 151	3	2	4	2	2	3	3	3	2	3	2	3
Respondent 152	1	2	1	4	3	4	4	4	4	3	5	5
Respondent 153	1	3	2	1	3	1	1	2	3	4	5	5
Respondent 154	1	3	2	3	2	3	3	2	5	3	2	6

Respondent 155	2	4	2	4	2	3	4	3	4	5	3	3
Respondent 156	3	3	3	2	1	4	2	4	3	2	5	5
Respondent 157	4	4	4	4	2	3	2	1	3	3	4	6
Respondent 158	1	1	2	2	2	5	4	3	3	4	3	3
Respondent 159	1	3	3	2	2	1	1	4	4	2	3	5
Respondent 160	5	4	2	4	3	3	3	2	3	4	5	5
Respondent 161	1	2	2	1	3	4	4	2	5	2	4	4
Respondent 162	2	2	3	3	3	2	2	2	2	4	1	3
Respondent 163	1	1	3	4	2	4	4	3	3	3	2	5
Respondent 164	2	2	4	1	2	2	3	4	4	5	6	3
Respondent 165	3	3	3	3	1	3	4	1	2	2	4	5
Respondent 166	2	4	1	4	2	3	2	3	4	2	3	5
Respondent 167	2	1	2	2	2	4	2	3	2	4	5	6
Respondent 168	3	2	3	2	2	3	4	4	1	1	3	3
Respondent 169	1	3	4	4	3	5	1	2	3	3	4	5
Respondent 170	2	2	1	1	3	1	3	2	4	4	2	5
Respondent 171	1	2	2	3	2	3	4	4	2	2	5	4
Respondent 172	2	3	2	4	2	4	2	1	4	2	5	3
Respondent 173	2	3	2	2	1	2	2	2	3	4	2	5
Respondent 174	3	4	3	4	2	3	3	4	3	1	3	5
Respondent 175	3	3	4	1	2	1	4	1	2	3	5	3
Respondent 176	3	1	2	3	2	2	1	3	3	4	4	4
Respondent 177	1	2	3	4	3	3	3	4	4	1	3	2
Respondent 178	1	3	2	2	3	4	4	2	2	3	5	4
Respondent 179	1	4	2	4	2	1	2	2	4	2	5	3
Respondent 180	2	1	3	2	2	3	2	3	2	4	4	3
Respondent 181	3	2	3	2	1	3	4	4	4	1	3	2
Respondent 182	1	2	4	4	2	4	1	1	3	2	5	3
Respondent 183	2	2	3	1	2	3	3	3	5	4	6	4
Respondent 184	1	3	1	3	2	5	4	4	4	3	4	2
Respondent 185	3	4	2	4	3	1	4	2	3	3	3	4
Respondent 186	1	3	3	1	3	3	1	2	3	3	5	2
Respondent 187	2	2	4	3	3	4	3	1	3	4	2	4
Respondent 188	1	3	1	4	2	2	4	3	4	3	4	3
Respondent 189	2	2	2	2	2	4	2	3	3	5	3	5
Respondent 190	3	2	2	2	1	2	4	4	5	2	5	4
Respondent 191	2	3	2	4	2	3	3	2	2	3	4	3
Respondent 192	2	3	3	1	2	3	4	2	3	4	1	4
Respondent 193	3	4	4	3	2	4	2	4	4	2	2	5
Respondent 194	1	3	2	4	1	3	2	1	2	4	6	4
Respondent 195	2	5	3	2	2	5	4	2	4	2	4	3
Respondent 196	1	2	2	4	2	1	1	4	2	4	3	5
Respondent 197	2	3	2	1	2	3	3	1	1	3	5	5
Respondent 198	2	4	3	3	3	4	4	3	3	4	3	6
Respondent 199	3	1	3	4	4	2	2	4	4	3	4	3
Respondent 200	3	2	4	2	1	3	2	2	2	5	2	5
Respondent 201	3	1	3	4	2	1	3	2	4	2	5	5
Respondent 202	1	2	1	2	3	2	4	3	2	3	5	4
Respondent 203	1	3	2	2	1	3	1	4	4	4	2	6
Respondent 204	1	4	3	4	2	4	3	1	2	2	3	3
Respondent 205	2	1	4	1	2	1	4	3	4	4	5	2
Respondent 206	3	2	1	3	2	2	2	4	3	2	4	3
Respondent 207	4	3	2	4	3	3	2	2	5	4	3	4
Respondent 208	1	2	2	1	4	1	4	2	4	3	5	2

Respondent 209	1	2	2	3	1	2	1	2	3	5	5	4
Respondent 210	5	3	3	4	2	1	3	3	3	4	4	2
Respondent 211	1	3	4	2	3	3	4	4	3	3	3	3
Respondent 212	2	4	4	2	1	1	4	1	4	3	5	3
Respondent 213	1	3	2	4	2	2	1	3	3	3	5	4
Respondent 214	2	5	3	1	2	1	3	3	5	4	4	2
Respondent 215	3	1	2	3	2	2	4	4	2	4	3	2
Respondent 216	2	3	2	4	3	4	2	2	3	3	5	3
Respondent 217	2	4	3	2	4	3	4	2	4	2	2	4
Respondent 218	3	1	3	4	2	5	3	3	2	2	4	2
Respondent 219	1	2	4	4	2	1	4	4	4	4	3	4
Respondent 220	2	1	3	1	3	3	2	1	2	1	3	3
Respondent 221	1	2	1	3	3	4	2	3	1	3	3	4
Respondent 222	2	3	2	4	3	2	4	4	3	4	4	2
Respondent 223	2	4	3	2	2	4	1	2	4	2	3	4
Respondent 224	3	1	4	4	2	3	3	2	2	2	2	3
Respondent 225	3	2	1	1	1	2	4	1	4	4	2	3
Respondent 226	3	3	2	3	2	1	2	3	3	1	3	2
Respondent 227	1	2	2	4	2	3	2	3	4	3	4	3
Respondent 228	1	2	2	2	2	1	3	4	2	4	2	4
Respondent 229	1	3	3	4	1	2	4	2	2	3	4	2
Respondent 230	2	3	4	2	2	1	1	2	4	5	2	4
Respondent 231	3	4	4	2	2	4	3	4	1	2	4	2
Respondent 232	4	3	2	4	2	3	4	1	3	3	3	4
Respondent 233	1	5	3	1	3	5	2	2	4	4	4	3
Respondent 234	4	1	2	3	4	1	2	4	2	2	3	5
Respondent 235	1	3	2	4	1	3	4	1	2	4	5	6
Respondent 236	1	4	3	1	2	4	1	3	4	2	2	3
Respondent 237	2	1	3	3	3	2	3	4	1	4	3	5
Respondent 238	1	2	4	4	1	3	4	2	3	3	4	5
Respondent 239	2	1	3	2	2	1	4	2	4	5	2	4
Respondent 240	3	2	1	2	2	2	1	3	1	4	4	3
Respondent 241	2	3	2	4	2	3	3	4	3	3	2	5
Respondent 242	2	4	3	1	3	4	4	1	3	3	3	5
Respondent 243	3	1	4	3	2	1	2	3	2	3	3	3
Respondent 244	1	2	1	4	2	3	4	4	3	4	4	4
Respondent 245	2	3	2	4	3	3	3	2	4	4	3	2
Respondent 246	1	2	2	1	3	4	4	2	2	3	5	4
Respondent 247	2	2	2	3	3	3	2	2	4	2	2	3
Respondent 248	2	3	3	4	2	5	2	3	2	2	3	3
Respondent 249	3	3	4	2	2	1	4	4	4	4	4	2
Respondent 250	3	4	4	4	1	3	1	3	2	1	2	3
Respondent 251	3	3	2	1	2	4	3	4	4	3	4	4
Respondent 252	1	5	3	3	2	2	4	1	3	4	2	2
Respondent 253	1	1	2	4	2	4	2	3	5	2	4	4
Respondent 254	1	3	2	2	1	2	2	4	4	2	3	2
Respondent 255	2	4	3	4	2	4	3	2	3	4	4	4
Respondent 256	3	1	3	2	2	3	4	2	3	1	3	3
Respondent 257	4	2	4	2	2	5	1	1	3	3	5	5
Respondent 258	1	2	3	4	3	1	3	3	4	4	2	6
Respondent 259	1	2	1	1	4	3	4	3	3	3	3	3
Respondent 260	5	3	2	3	1	4	2	4	5	5	4	5
Respondent 261	1	4	3	4	2	2	2	2	2	2	2	5
Respondent 262	2	1	4	1	3	3	4	2	3	3	4	4

Respondent 263	1	2	1	3	1	1	1	4	4	4	2	3
Respondent 264	2	3	2	4	2	2	3	1	2	2	3	5
Respondent 265	3	2	2	2	2	3	4	2	4	4	3	5
Respondent 266	2	2	2	2	2	4	4	4	2	2	4	3
Respondent 267	2	3	3	4	3	1	1	1	1	4	3	4
Respondent 268	3	3	4	1	2	3	3	3	3	3	5	5
Respondent 269	1	4	2	3	2	3	4	4	4	5	2	4
Respondent 270	2	3	3	4	3	4	2	2	2	4	3	3
Respondent 271	1	5	2	4	3	3	4	2	4	4	4	5
Respondent 272	2	1	2	1	3	5	3	3	3	3	2	5
Respondent 273	2	3	3	3	2	1	4	4	4	3	4	6
Respondent 274	3	4	3	4	2	3	2	1	2	3	2	5
Respondent 275	3	1	4	2	1	4	2	3	2	4	4	6
Respondent 276	3	2	3	4	2	2	4	4	4	3	3	2
Respondent 277	1	3	1	1	2	4	1	2	1	5	4	2
Respondent 278	1	2	2	3	2	2	3	2	3	2	3	3
Respondent 279	1	3	3	4	1	4	4	2	4	3	5	4
Respondent 280	2	4	4	2	2	3	2	3	2	4	2	1
Respondent 281	3	1	1	4	2	5	2	4	2	2	3	2
Respondent 282	4	2	2	2	2	1	3	3	4	4	4	3
Respondent 283	1	3	2	2	3	3	4	4	1	2	2	1
Respondent 284	1	2	2	4	4	4	1	1	3	4	4	2
Respondent 285	5	2	3	1	1	2	3	3	4	3	2	2
Respondent 286	1	3	2	3	2	3	4	4	1	4	3	2
Respondent 287	2	3	3	4	3	1	2	2	3	3	3	3
Respondent 288	1	4	4	1	1	2	2	2	3	5	4	2
Respondent 289	2	3	2	3	2	3	4	1	2	2	2	2
Respondent 290	3	3	3	4	2	4	1	3	4	3	4	3
Respondent 291	2	1	2	2	2	1	3	3	1	4	3	3
Respondent 292	2	3	2	2	3	3	4	4	3	2	5	3
Respondent 293	3	4	3	4	2	3	4	2	4	4	3	4
Respondent 294	1	1	3	1	2	4	1	2	2	2	4	3
Respondent 295	2	2	4	3	3	3	3	4	2	4	2	5
Respondent 296	1	1	3	4	3	5	4	1	3	3	5	5
Respondent 297	2	2	1	4	3	1	2	2	4	5	5	6
Respondent 298	2	3	2	1	2	3	4	4	1	4	2	5
Respondent 299	3	1	3	3	2	4	3	1	3	4	3	6
Respondent 300	3	1	4	4	1	2	4	3	4	3	2	3
Respondent 301	3	2	1	2	2	4	2	4	2	3	4	5
Respondent 302	1	3	2	4	2	2	2	2	2	3	3	5
Respondent 303	1	2	2	1	2	4	4	2	2	4	2	4
Respondent 304	1	2	2	3	1	3	1	3	3	3	5	3
Respondent 305	2	3	3	4	2	5	3	4	4	5	4	5
Respondent 306	3	3	2	2	2	1	4	1	3	2	3	5
Respondent 307	4	4	3	4	2	3	2	3	4	3	5	6
Respondent 308	1	3	4	2	3	4	2	4	1	4	6	5
Respondent 309	1	3	2	2	4	2	3	2	3	2	4	6
Respondent 310	5	1	3	4	1	3	4	2	2	4	3	3
Respondent 311	1	3	2	1	2	1	1	2	4	2	2	5
Respondent 312	2	4	2	3	3	2	3	3	1	4	6	5
Respondent 313	1	1	3	4	1	3	4	4	3	3	4	4
Respondent 314	2	2	3	1	2	4	2	3	4	4	3	5
Respondent 315	3	1	4	3	2	1	2	4	2	3	5	6
Respondent 316	2	2	3	4	2	3	4	1	2	5	3	5

Respondent 317	2	3	1	2	3	3	1	3	3	2	4	4
Respondent 318	3	4	2	2	2	4	3	4	4	3	2	3
Respondent 319	1	1	3	4	2	3	4	2	1	4	5	5
Respondent 320	2	2	4	1	3	5	4	2	3	2	5	4
Respondent 321	1	3	1	3	3	1	1	1	4	4	2	3
Respondent 322	2	2	2	4	3	3	3	3	2	2	3	5
Respondent 323	2	2	2	4	2	4	4	3	2	4	5	3
Respondent 324	3	3	2	1	2	2	2	4	2	3	4	4
Respondent 325	3	3	3	3	1	4	4	2	3	5	3	2
Respondent 326	3	1	2	4	2	2	3	2	4	4	5	5
Respondent 327	1	3	3	2	2	4	4	4	3	4	5	5
Respondent 328	1	5	4	4	2	3	2	1	4	3	4	2
Respondent 329	1	1	2	1	1	5	2	2	1	3	3	3
Respondent 330	2	3	3	3	2	1	4	4	3	3	5	2
Respondent 331	3	4	2	3	2	3	1	1	2	4	6	4
Respondent 332	4	1	2	4	2	4	3	3	4	3	4	3
Respondent 333	1	2	3	4	3	2	4	4	1	5	3	2
Respondent 334	1	2	3	1	4	3	2	2	3	2	5	5
Respondent 335	5	2	4	3	1	1	2	2	4	3	6	4
Respondent 336	1	3	3	4	2	2	3	3	2	4	4	3
Respondent 337	2	4	1	2	3	1	4	4	2	2	3	5
Respondent 338	1	2	2	4	1	3	1	1	3	4	5	6
Respondent 339	2	2	3	1	2	1	3	3	4	2	3	4
Respondent 340	3	3	4	3	2	2	4	4	1	4	4	3
Respondent 341	2	2	1	4	2	1	2	3	3	3	2	2
Respondent 342	2	2	2	2	3	2	2	4	4	4	5	6
Respondent 343	3	2	2	4	2	4	4	2	2	3	5	4
Respondent 344	1	3	2	2	2	3	1	2	2	5	2	4
Respondent 345	2	3	3	2	3	5	3	4	2	2	3	3
Respondent 346	1	3	2	4	3	1	4	1	3	3	5	5
Respondent 347	2	5	3	1	3	3	4	2	4	4	4	3
Respondent 348	2	1	4	3	2	4	1	4	3	2	3	4
Respondent 349	3	3	2	4	2	2	3	1	4	4	5	2
Respondent 350	1	4	3	3	1	4	4	3	1	2	5	5
Respondent 351	3	1	2	4	2	3	2	4	3	4	4	5
Respondent 352	1	2	2	4	2	2	4	2	2	3	3	2
Respondent 353	1	1	3	1	2	1	3	2	4	5	5	3
Respondent 354	1	2	3	3	1	3	4	3	1	4	3	2
Respondent 355	2	3	4	4	2	1	2	4	2	3	6	4
Respondent 356	3	4	3	2	2	3	2	1	4	3	3	3
Respondent 357	4	1	1	4	2	1	4	3	3	3	5	2
Respondent 358	1	2	2	1	3	2	1	4	4	4	4	5
Respondent 359	1	3	3	3	4	1	3	2	2	3	4	4
Respondent 360	1	2	4	4	1	3	4	2	2	5	3	3
Respondent 361	1	2	1	2	2	1	2	2	4	2	4	5
Respondent 362	2	2	2	4	3	2	2	3	1	3	3	6
Respondent 363	1	3	2	2	1	1	3	4	3	4	4	4
Respondent 364	2	4	2	2	2	2	4	3	4	2	2	3
Respondent 365	3	3	3	4	2	4	1	4	2	4	5	2
Respondent 366	2	2	2	1	2	3	3	1	2	2	4	6
Respondent 367	1	1	3	3	3	5	4	3	4	4	2	4
Respondent 368	3	3	4	4	2	1	2	4	1	3	3	4
Respondent 369	1	4	2	3	2	3	2	3	3	4	5	3
Respondent 370	2	1	3	4	3	4	4	4	4	3	4	3

Respondent 371	1	2	2	4	3	2	1	2	1	5	3	5
Respondent 372	2	1	2	1	3	4	3	2	3	2	5	4
Respondent 373	1	2	3	3	2	3	4	4	3	3	5	4
Respondent 374	3	3	3	4	2	2	1	1	2	4	4	3
Respondent 375	1	4	4	2	1	1	3	2	4	2	3	4
Respondent 376	3	1	3	4	2	3	4	4	1	4	5	3
Respondent 377	1	1	1	1	2	1	2	1	3	2	6	4
Respondent 378	1	3	2	3	2	3	4	3	4	4	4	2
Respondent 379	1	2	3	4	3	1	3	4	2	3	2	5
Respondent 380	2	2	4	2	4	2	4	2	2	3	3	4
Respondent 381	1	3	1	4	1	1	2	2	3	3	6	2
Respondent 382	4	3	2	2	2	3	2	3	4	4	4	3
Respondent 383	1	4	2	2	3	1	4	4	1	3	3	5
Respondent 384	1	3	2	4	1	2	1	1	3	5	5	4
Respondent 385	4	2	3	1	2	1	3	3	4	2	3	3
Respondent 386	1	1	2	3	2	2	4	4	2	3	4	5
Respondent 387	2	3	2	4	2	4	2	2	2	4	2	2
Respondent 388	1	4	3	3	3	3	2	2	2	2	5	2
Respondent 389	2	1	2	4	2	3	3	2	4	4	6	3
Respondent 390	3	2	2	4	2	3	4	3	3	2	2	4
Respondent 391	2	1	3	1	1	4	1	4	4	4	3	1
Respondent 393	2	2	3	3	2	3	3	3	2	3	5	2
Respondent 394	1	3	4	4	2	5	4	4	2	4	4	3
Respondent 395	1	4	3	2	2	1	2	1	4	3	3	1
Respondent 396	2	1	1	4	3	3	2	3	1	5	3	2
Respondent 397	1	2	2	1	4	4	2	4	3	2	5	2
Respondent 398	2	3	3	3	1	2	3	3	4	3	4	2
Respondent 399	2	2	4	4	2	4	4	4	2	4	3	3
Respondent 400	3	2	1	2	3	2	1	2	2	2	4	2
Respondent 401	1	3	2	4	1	4	3	2	4	4	4	2
Respondent 402	3	3	2	2	2	3	4	4	1	2	4	3
Respondent 403	1	4	2	2	2	5	2	1	3	4	3	3
Respondent 404	1	3	3	4	2	1	2	2	4	2	5	3
Respondent 405	1	2	2	1	3	3	4	4	1	4	6	2
Respondent 406	2	1	3	3	2	4	1	2	3	3	4	3
Respondent 407	1	3	4	4	2	3	3	2	3	4	3	4
Respondent 408	4	4	2	3	1	3	4	2	2	2	5	2
Respondent 409	1	1	3	4	2	4	4	3	4	2	3	4
Respondent 410	3	2	2	4	2	3	1	2	1	4	4	2
Respondent 411	3	1	2	1	2	5	3	2	3	2	2	3
Respondent 412	1	2	2	3	3	1	4	3	4	4	5	3
Respondent 413	2	3	3	4	4	3	2	2	2	3	5	4
Respondent 414	1	4	2	2	1	4	4	2	2	4	2	2
Respondent 415	2	1	2	4	2	2	3	3	3	2	3	2
Respondent 416	3	2	3	1	3	4	4	3	4	2	5	3
Respondent 417	2	3	3	3	1	2	2	4	2	4	4	4

Response to questionnaire (13-24)

	Fri1	Fri2	Fri3	Fri4	Con1	Con2	Con3	Con4	Ope1	Ope2	Ope3	Ope4
Respondent 1	3	2	3	3	2	1	4	1	2	2	3	2
Respondent 2	1	2	4	2	2	1	1	3	2	2	4	2
Respondent 3	2	3	3	3	1	2	2	2	3	3	1	3
Respondent 4	1	3	5	4	2	3	3	3	1	3	2	3
Respondent 5	2	4	1	3	2	2	2	2	2	4	2	4
Respondent 6	2	3	3	4	2	2	3	3	1	3	2	3
Respondent 7	3	5	4	2	3	3	4	4	2	5	3	4
Respondent 8	3	1	1	3	1	3	3	3	2	1	4	1
Respondent 9	3	3	2	2	1	4	4	4	3	3	1	3
Respondent 10	1	4	2	2	2	3	1	4	3	4	2	4
Respondent 11	1	1	2	3	2	5	2	3	3	1	3	2
Respondent 12	1	2	3	4	3	1	2	5	1	2	2	2
Respondent 13	2	2	4	3	2	3	2	3	1	2	2	4
Respondent 14	3	2	1	1	1	4	3	4	1	2	3	1
Respondent 15	4	3	2	5	2	1	4	2	2	3	3	3
Respondent 16	1	4	3	3	2	2	1	3	3	4	4	4
Respondent 17	1	1	2	4	3	1	3	4	4	1	3	2
Respondent 18	5	2	3	3	2	2	5	1	1	2	4	2
Respondent 19	1	3	4	2	2	3	3	3	1	3	1	4
Respondent 20	2	2	3	4	3	4	4	2	5	2	3	1
Respondent 21	1	2	4	3	2	1	4	1	1	2	4	3
Respondent 22	2	3	1	2	2	1	1	3	2	3	2	4
Respondent 23	3	3	2	3	1	2	2	2	1	3	2	1
Respondent 24	2	4	2	4	2	3	3	3	2	4	1	3
Respondent 25	2	3	2	3	2	2	2	2	3	3	2	4
Respondent 26	3	4	3	4	3	3	3	1	2	4	3	2
Respondent 27	1	1	4	2	3	5	4	2	2	1	4	2
Respondent 28	2	3	1	3	4	1	3	2	3	3	1	4
Respondent 29	1	4	2	2	3	3	4	2	1	4	2	1
Respondent 30	2	2	3	2	5	4	2	3	2	2	3	3
Respondent 31	2	2	2	3	1	1	3	1	1	2	2	4
Respondent 32	3	1	3	4	3	2	2	1	2	1	2	2
Respondent 33	3	2	4	3	4	2	2	2	2	2	3	4
Respondent 34	3	3	3	3	1	2	3	2	2	2	4	2
Respondent 35	1	4	4	5	2	3	4	3	2	2	3	3
Respondent 36	1	1	1	3	2	4	3	2	1	2	3	3
Respondent 37	1	2	2	4	2	1	1	1	2	3	5	4
Respondent 38	2	3	2	3	3	2	5	2	3	4	3	3
Respondent 39	3	2	2	2	4	3	3	2	4	1	4	3
Respondent 40	4	2	3	3	1	2	4	3	1	2	3	3
Respondent 41	1	3	4	3	2	3	3	2	2	2	4	2
Respondent 42	1	3	1	2	3	4	2	2	2	2	3	3
Respondent 43	5	4	2	3	2	3	4	3	1	2	3	3
Respondent 44	1	3	3	4	2	4	3	2	2	1	4	1
Respondent 45	3	1	2	3	3	1	2	2	3	3	1	3
Respondent 46	1	2	3	4	3	2	3	1	3	4	2	4
Respondent 47	2	3	5	2	4	2	4	2	3	1	3	2
Respondent 48	1	4	3	3	3	2	3	2	1	2	2	2
Respondent 49	2	1	4	2	3	3	3	1	1	2	2	4

Respondent 50	2	2	1	2	3	5	4	2	1	2	3	1
Respondent 51	3	2	2	3	4	1	3	2	2	3	3	3
Respondent 52	3	2	2	4	2	2	1	3	3	4	4	4
Respondent 53	2	3	2	3	3	1	3	4	4	1	3	2
Respondent 54	2	4	3	3	2	2	5	1	2	2	3	2
Respondent 55	3	3	4	5	2	3	3	3	2	2	4	2
Respondent 56	3	2	1	3	3	4	4	2	3	3	1	3
Respondent 57	3	3	2	4	2	1	4	1	1	3	2	3
Respondent 58	1	2	3	3	2	1	1	3	2	4	2	4
Respondent 59	1	2	2	2	1	2	2	2	1	3	2	3
Respondent 60	1	3	3	3	2	3	3	3	2	5	3	4
Respondent 61	2	2	4	3	2	2	2	2	2	1	4	1
Respondent 62	3	3	3	2	3	3	3	1	3	3	1	3
Respondent 63	4	2	5	3	3	5	4	2	3	4	2	4
Respondent 64	1	2	1	4	4	1	3	2	3	1	3	2
Respondent 65	1	3	2	3	3	3	4	2	1	2	2	2
Respondent 66	5	3	2	4	5	4	2	3	1	2	2	4
Respondent 67	1	4	2	2	1	1	3	1	1	2	3	1
Respondent 68	3	3	3	3	3	2	2	1	2	3	3	3
Respondent 69	1	5	4	2	4	2	2	2	3	4	4	4
Respondent 70	2	1	1	2	1	2	3	2	4	1	3	2
Respondent 71	2	3	2	3	2	3	4	3	1	2	4	2
Respondent 72	3	4	3	4	2	4	3	2	1	3	1	4
Respondent 73	3	1	2	3	2	2	1	3	5	2	3	1
Respondent 74	3	2	3	3	3	1	3	4	1	2	4	3
Respondent 75	1	2	4	5	2	2	5	1	2	3	2	4
Respondent 76	1	2	3	3	2	3	3	3	1	3	2	1
Respondent 77	1	3	4	4	3	4	4	2	2	4	1	3
Respondent 78	2	4	1	3	5	4	2	3	3	3	2	4
Respondent 79	3	1	2	2	1	1	3	1	2	4	3	2
Respondent 80	4	2	2	3	3	2	2	1	2	1	4	2
Respondent 81	1	3	2	3	4	2	2	2	3	3	1	4
Respondent 82	1	2	3	2	1	2	3	2	1	4	2	1
Respondent 83	5	2	4	3	2	3	4	3	2	2	3	3
Respondent 84	1	3	1	4	2	4	3	2	1	2	2	4
Respondent 85	3	3	2	3	2	1	1	1	2	1	2	2
Respondent 86	1	4	3	4	3	2	5	2	2	2	3	4
Respondent 87	2	3	3	2	4	3	3	2	3	3	3	1
Respondent 88	2	4	5	3	1	2	4	3	3	4	4	3
Respondent 89	3	1	3	2	2	3	3	2	3	1	3	4
Respondent 90	3	3	4	2	3	4	2	2	2	2	3	2
Respondent 91	2	4	1	3	2	3	4	3	2	2	4	2
Respondent 92	3	2	2	4	2	4	3	2	2	2	3	3
Respondent 93	2	2	2	3	3	1	2	2	1	2	3	3
Respondent 94	2	1	2	3	3	2	3	1	2	3	5	4
Respondent 95	3	2	3	5	4	2	4	2	3	4	3	3
Respondent 96	1	3	4	3	3	2	3	2	4	1	4	3
Respondent 97	2	4	1	4	3	3	3	1	1	2	3	3
Respondent 98	2	1	2	3	3	5	4	2	3	3	1	3
Respondent 99	3	2	3	2	5	4	2	3	3	4	2	4
Respondent 100	2	3	2	3	1	1	3	1	3	1	3	2
Respondent 101	2	2	3	3	3	2	2	1	1	2	2	2
Respondent 102	3	2	4	2	4	2	2	2	1	2	2	4
Respondent 103	1	3	3	3	1	2	3	2	1	2	3	1

Respondent 104	2	3	5	4	2	3	4	3	2	3	3	3
Respondent 105	2	4	1	3	2	4	3	2	3	4	4	4
Respondent 106	3	3	2	4	2	1	1	1	4	1	3	2
Respondent 107	3	1	2	4	3	2	5	2	1	2	4	2
Respondent 108	3	1	2	2	4	3	3	2	1	3	1	4
Respondent 109	1	2	3	3	1	2	4	3	5	2	3	1
Respondent 110	1	3	4	2	2	3	3	2	2	2	4	2
Respondent 111	1	2	1	2	3	4	2	2	3	3	1	3
Respondent 112	2	2	2	3	2	3	4	3	1	3	2	3
Respondent 113	3	3	3	4	2	4	3	2	2	4	2	4
Respondent 114	4	3	2	3	3	1	2	2	1	3	2	3
Respondent 115	1	4	3	1	3	2	3	1	2	5	3	4
Respondent 116	1	3	4	5	4	2	4	2	2	1	4	1
Respondent 117	5	5	3	3	3	2	3	2	3	3	1	3
Respondent 118	1	1	4	4	3	3	3	1	3	4	2	4
Respondent 119	3	3	1	3	3	5	4	2	3	1	3	2
Respondent 120	1	4	2	2	5	4	2	3	1	2	2	2
Respondent 121	2	1	2	4	1	1	3	1	1	2	2	4
Respondent 122	2	2	2	4	3	2	2	1	1	2	3	1
Respondent 123	3	1	3	2	4	2	2	2	2	3	3	3
Respondent 124	3	2	4	3	1	2	3	2	3	4	4	4
Respondent 125	3	3	1	2	2	3	4	3	4	1	3	2
Respondent 126	2	4	3	2	2	4	3	2	1	2	4	2
Respondent 127	3	1	5	3	2	1	1	1	1	3	1	4
Respondent 128	2	2	3	4	3	2	5	2	5	2	3	1
Respondent 129	2	3	4	3	5	2	4	3	1	2	4	3
Respondent 130	3	2	1	1	1	3	1	4	2	3	2	4
Respondent 131	4	2	2	5	3	3	2	3	1	3	2	1
Respondent 132	1	3	2	3	1	4	3	4	2	4	1	3
Respondent 133	1	3	2	4	2	3	3	2	3	3	2	4
Respondent 134	2	4	3	3	2	4	5	3	2	4	3	2
Respondent 135	3	3	4	2	3	1	3	2	2	1	4	2
Respondent 136	2	5	1	4	3	3	4	2	3	3	1	4
Respondent 137	2	1	2	4	2	4	1	3	1	4	2	1
Respondent 138	3	3	3	2	3	2	2	4	2	2	3	3
Respondent 139	1	2	2	3	2	2	2	3	1	2	2	4
Respondent 140	2	2	3	2	2	1	2	3	2	1	2	2
Respondent 141	2	3	4	2	3	2	3	5	2	2	3	4
Respondent 142	3	3	3	3	1	3	4	3	3	3	3	1
Respondent 143	2	4	5	4	2	4	1	4	3	4	4	3
Respondent 144	2	3	1	3	2	1	2	3	3	1	3	4
Respondent 145	3	5	2	1	3	2	3	2	1	2	1	2
Respondent 146	1	1	2	5	2	3	2	3	1	3	2	2
Respondent 147	2	3	2	3	2	2	3	3	1	2	3	4
Respondent 148	2	4	3	4	3	2	4	2	2	2	4	1
Respondent 149	3	1	4	3	5	2	4	3	2	2	4	2
Respondent 150	2	2	1	2	1	3	1	4	3	3	1	3
Respondent 151	2	2	2	4	3	3	2	3	1	3	2	3
Respondent 152	3	2	3	4	1	4	3	4	2	4	2	4
Respondent 153	1	3	2	2	2	3	3	2	1	3	2	3
Respondent 154	2	4	3	3	2	4	5	3	2	5	3	4
Respondent 155	2	1	4	2	3	1	3	2	2	1	4	1
Respondent 156	2	2	3	2	3	3	4	2	3	3	1	3
Respondent 157	2	3	4	3	2	4	1	3	3	4	2	4

Respondent 158	2	2	1	4	3	2	2	4	3	1	3	2
Respondent 159	3	2	2	3	2	2	2	3	1	2	2	2
Respondent 160	1	3	2	2	2	1	2	3	1	2	2	4
Respondent 161	2	3	2	3	3	2	3	5	1	2	3	1
Respondent 162	2	4	3	4	1	3	4	3	2	3	3	3
Respondent 163	3	3	4	1	2	4	1	4	3	4	4	4
Respondent 164	2	4	1	3	2	1	2	3	4	1	3	2
Respondent 165	2	1	3	5	3	2	3	2	1	2	4	2
Respondent 166	3	3	5	3	2	3	2	3	1	3	1	4
Respondent 167	1	4	3	4	2	2	3	3	5	2	3	1
Respondent 168	2	2	4	2	3	2	4	2	1	2	4	3
Respondent 169	2	2	1	3	5	2	4	3	2	3	2	4
Respondent 170	3	1	2	4	1	3	1	4	1	3	2	1
Respondent 171	2	2	2	1	3	3	2	3	2	4	1	3
Respondent 172	2	3	2	3	1	4	3	4	3	3	2	4
Respondent 173	3	4	3	5	2	3	3	2	2	4	3	2
Respondent 174	1	1	4	3	2	4	5	3	2	1	4	2
Respondent 175	2	2	1	4	3	1	3	2	3	3	1	4
Respondent 176	2	3	2	2	3	3	4	2	1	4	2	1
Respondent 177	3	2	3	3	2	4	1	3	2	2	3	3
Respondent 178	2	2	2	4	3	2	2	4	1	2	2	4
Respondent 179	2	3	3	1	2	2	2	3	2	1	2	2
Respondent 180	3	3	4	3	2	1	2	3	2	2	3	4
Respondent 181	1	4	3	5	3	2	3	5	3	3	3	1
Respondent 182	2	3	5	3	1	3	4	3	3	4	4	3
Respondent 183	2	1	1	4	2	4	1	4	3	1	3	4
Respondent 184	1	2	2	2	2	1	2	3	1	2	1	2
Respondent 185	2	3	2	3	3	2	3	2	1	3	2	2
Respondent 186	2	4	2	4	2	3	2	3	1	2	3	4
Respondent 187	3	1	3	1	2	2	3	3	2	2	4	1
Respondent 188	1	2	4	3	3	2	4	2	2	2	4	2
Respondent 189	2	2	1	5	5	2	4	3	3	3	1	3
Respondent 190	2	2	2	3	1	3	1	4	1	3	2	3
Respondent 191	1	3	3	4	3	3	2	3	2	4	2	4
Respondent 192	2	4	2	2	1	4	3	4	1	3	2	3
Respondent 193	2	3	3	3	2	3	3	2	2	5	3	4
Respondent 194	3	2	4	4	2	4	5	3	2	1	4	1
Respondent 195	1	3	3	1	3	1	3	2	4	2	3	2
Respondent 196	2	2	4	3	3	3	4	2	1	2	2	3
Respondent 197	2	2	1	2	2	4	1	3	3	3	2	4
Respondent 198	3	3	2	3	3	2	2	4	5	5	2	4
Respondent 199	2	2	2	2	2	2	2	3	3	1	3	1
Respondent 200	2	3	2	3	2	1	2	3	4	3	3	2
Respondent 201	3	2	3	4	3	2	3	5	2	1	4	3
Respondent 202	1	2	4	3	1	3	4	3	3	2	3	3
Respondent 203	2	3	1	4	2	4	1	4	4	2	4	5
Respondent 204	2	3	3	2	2	1	2	3	1	3	1	3
Respondent 205	3	4	5	3	3	2	3	2	3	3	3	4
Respondent 206	2	3	3	2	2	3	2	3	2	2	4	1
Respondent 207	2	5	4	3	2	2	3	3	3	3	2	2
Respondent 208	3	1	1	4	3	2	4	2	2	2	2	2
Respondent 209	1	3	2	3	5	2	4	3	3	2	1	2
Respondent 210	2	4	2	4	1	3	1	4	4	3	2	3
Respondent 211	2	1	2	3	3	3	2	3	3	1	3	4

Respondent 212	3	2	3	2	1	4	3	4	4	2	4	1
Respondent 213	2	2	4	2	2	3	3	2	2	2	1	2
Respondent 214	2	2	1	3	2	4	5	3	3	3	1	4
Respondent 215	1	3	2	3	3	1	3	2	1	4	2	1
Respondent 216	2	4	3	4	3	3	4	2	2	2	3	3
Respondent 217	2	1	2	3	2	4	1	3	1	2	2	4
Respondent 218	3	2	3	5	3	2	2	4	2	1	2	2
Respondent 219	2	3	4	2	2	2	2	3	2	2	3	4
Respondent 220	2	2	3	3	2	1	2	3	3	3	3	1
Respondent 221	3	2	5	4	3	2	3	5	3	4	4	3
Respondent 222	1	3	1	1	1	3	4	3	3	1	3	4
Respondent 223	2	3	2	3	2	4	1	4	3	3	2	4
Respondent 224	2	4	2	2	2	1	2	3	5	5	2	4
Respondent 225	3	3	2	3	3	2	3	2	3	1	3	1
Respondent 226	2	4	3	2	2	3	2	3	4	3	3	2
Respondent 227	2	1	4	3	2	2	3	3	2	1	4	3
Respondent 228	3	3	1	4	3	2	4	2	3	2	3	3
Respondent 229	1	4	2	3	5	2	4	3	4	2	4	5
Respondent 230	2	2	3	4	3	2	3	3	1	3	1	3
Respondent 231	2	2	2	4	2	2	2	2	3	3	3	4
Respondent 232	3	1	3	1	3	2	3	2	2	2	4	1
Respondent 233	2	2	4	3	2	3	4	3	3	3	2	2
Respondent 234	1	3	3	5	2	4	3	1	2	2	2	2
Respondent 235	3	4	4	3	3	1	4	2	3	2	1	2
Respondent 236	1	1	1	4	3	3	2	2	4	3	2	3
Respondent 237	1	2	2	2	4	5	3	3	3	1	3	4
Respondent 238	2	3	2	3	3	3	2	2	4	2	4	1
Respondent 239	2	2	2	4	5	4	3	2	2	2	1	2
Respondent 240	3	2	3	1	1	1	4	3	3	3	1	4
Respondent 241	2	3	4	3	3	2	3	5	1	4	2	1
Respondent 242	2	3	1	2	4	2	4	1	2	2	3	3
Respondent 243	3	4	3	3	1	2	3	3	1	2	2	4
Respondent 244	1	3	5	2	2	3	2	1	2	1	2	2
Respondent 245	2	1	3	3	2	4	2	2	2	2	3	4
Respondent 246	2	1	4	2	2	1	3	2	3	3	3	1
Respondent 247	3	2	1	2	3	2	3	3	3	4	4	3
Respondent 248	2	3	2	3	4	3	4	3	3	1	3	4
Respondent 249	2	2	2	3	1	2	3	2	3	3	2	4
Respondent 250	3	2	2	4	3	2	3	3	5	5	2	4
Respondent 251	1	3	3	3	2	2	2	2	3	1	3	1
Respondent 252	2	3	4	5	3	2	3	2	4	3	3	2
Respondent 253	2	4	1	3	2	3	4	3	2	1	4	3
Respondent 254	3	3	2	4	2	4	3	1	3	2	3	3
Respondent 255	2	5	3	2	3	1	4	2	4	2	4	5
Respondent 256	2	1	2	3	3	3	2	2	1	3	1	3
Respondent 257	3	3	3	4	4	5	3	3	3	3	3	4
Respondent 258	1	4	4	1	3	3	2	2	2	2	4	1
Respondent 259	1	1	3	3	5	4	3	2	3	3	2	2
Respondent 260	2	2	5	2	1	1	4	3	2	2	2	2
Respondent 261	2	1	1	3	3	2	3	5	3	2	1	2
Respondent 262	1	2	2	2	4	2	4	1	4	3	2	3
Respondent 263	2	3	2	3	1	2	3	3	3	1	3	4
Respondent 264	2	4	2	4	2	3	2	1	4	2	4	1
Respondent 265	3	1	3	3	2	4	2	2	2	2	1	2

Respondent 266	1	2	4	4	2	1	3	2	3	3	1	4
Respondent 267	2	3	1	4	3	2	3	3	1	4	2	1
Respondent 268	2	2	2	1	4	3	4	3	2	2	3	3
Respondent 269	3	2	3	3	1	2	3	2	2	2	2	2
Respondent 270	2	3	2	5	3	2	3	3	3	2	1	2
Respondent 271	2	3	3	3	2	2	2	2	4	3	2	3
Respondent 272	3	4	4	4	3	2	3	2	3	1	3	4
Respondent 273	1	3	3	2	2	3	4	3	4	2	4	1
Respondent 274	2	5	4	3	2	4	3	1	2	2	1	2
Respondent 275	2	1	1	4	3	1	4	2	3	3	1	4
Respondent 276	3	3	2	3	3	3	2	2	1	4	2	1
Respondent 277	1	2	2	2	4	5	3	3	2	2	3	3
Respondent 278	2	2	2	2	3	3	2	2	1	2	2	4
Respondent 279	2	3	3	3	5	4	3	2	2	1	2	2
Respondent 280	3	3	4	3	1	1	4	3	2	2	3	4
Respondent 281	2	4	1	4	3	2	3	5	3	3	3	1
Respondent 282	2	3	3	3	4	2	4	1	3	4	4	3
Respondent 283	3	5	5	5	1	2	3	3	3	1	3	4
Respondent 284	1	1	3	3	2	3	2	1	3	3	2	4
Respondent 285	1	3	4	2	2	4	2	2	5	5	2	4
Respondent 286	2	4	1	2	2	1	3	2	3	1	3	1
Respondent 287	2	1	2	3	3	2	3	3	4	3	3	2
Respondent 288	3	2	2	3	4	3	4	3	2	1	4	3
Respondent 289	2	2	2	5	1	2	3	2	3	2	3	3
Respondent 290	2	2	3	3	3	2	3	3	4	2	4	5
Respondent 291	1	3	4	4	2	2	2	2	1	3	1	3
Respondent 292	1	4	1	2	3	2	3	2	3	3	3	4
Respondent 293	2	1	2	3	2	3	4	3	2	2	4	1
Respondent 294	2	2	3	4	2	4	3	1	3	3	2	2
Respondent 295	3	3	2	1	3	1	4	2	2	2	2	2
Respondent 296	2	2	3	3	3	3	2	2	3	2	1	2
Respondent 297	2	2	4	2	4	5	3	3	4	3	2	3
Respondent 298	3	3	3	3	3	3	2	2	3	1	3	4
Respondent 299	1	3	5	2	5	4	3	2	4	2	4	1
Respondent 300	1	4	1	3	1	1	4	3	2	2	2	2
Respondent 301	2	3	2	2	3	2	3	5	3	2	1	2
Respondent 302	2	4	2	2	4	2	4	1	4	3	2	3
Respondent 303	3	1	2	3	1	2	3	3	3	1	3	4
Respondent 304	2	3	3	3	2	3	2	1	4	2	4	1
Respondent 305	2	4	4	4	2	4	2	2	2	2	1	2
Respondent 306	3	2	1	3	2	3	3	2	3	3	1	4
Respondent 307	1	2	2	5	3	2	3	3	1	4	2	1
Respondent 308	1	1	3	3	4	3	4	3	2	2	3	3
Respondent 309	2	2	2	4	3	3	3	2	1	2	2	4
Respondent 310	2	3	3	2	2	5	3	2	2	1	2	2
Respondent 311	1	4	4	3	3	3	2	2	2	2	3	4
Respondent 312	2	1	3	4	4	4	3	2	3	3	3	1
Respondent 313	2	2	4	3	3	2	2	3	3	4	4	3
Respondent 314	3	3	1	3	4	3	2	4	3	1	3	4
Respondent 315	1	2	2	2	2	4	3	2	3	3	2	4
Respondent 316	2	2	2	3	2	3	3	3	5	5	2	4
Respondent 317	2	3	2	2	2	2	4	5	3	1	3	1
Respondent 318	3	3	3	3	2	2	3	3	4	3	3	2
Respondent 319	2	4	4	4	3	3	5	4	2	1	4	3

Respondent 320	2	3	1	3	4	3	2	3	3	2	3	3
Respondent 321	3	1	3	4	2	4	3	2	4	2	4	5
Respondent 322	1	2	5	4	3	3	4	2	1	3	1	3
Respondent 323	1	3	3	3	5	5	1	2	3	3	3	4
Respondent 324	2	4	4	5	4	2	4	2	2	2	4	1
Respondent 325	2	1	1	3	3	2	3	3	3	3	2	2
Respondent 326	3	2	2	4	5	3	2	3	2	2	2	2
Respondent 327	2	2	2	2	3	4	3	4	3	2	1	2
Respondent 328	1	2	2	3	4	3	3	3	4	3	2	3
Respondent 329	3	3	3	4	2	2	5	3	3	1	3	4
Respondent 330	1	4	4	1	3	3	3	2	4	2	4	1
Respondent 331	1	3	1	3	4	4	4	3	2	2	2	2
Respondent 332	2	2	2	2	3	3	2	2	3	2	1	2
Respondent 333	2	3	3	3	3	4	3	2	4	3	2	3
Respondent 334	3	2	2	2	2	2	4	3	3	1	3	4
Respondent 335	2	2	3	3	3	2	3	3	4	2	4	1
Respondent 336	2	3	4	2	2	2	2	4	2	2	1	2
Respondent 337	1	2	3	2	3	2	2	3	3	3	1	4
Respondent 338	1	3	5	3	4	3	3	5	1	4	2	1
Respondent 339	2	2	1	3	3	4	3	2	2	2	3	3
Respondent 340	2	2	2	4	4	2	2	2	1	2	2	4
Respondent 341	3	3	2	3	4	3	3	3	2	1	2	2
Respondent 342	2	3	2	5	3	5	4	4	2	2	3	4
Respondent 343	2	4	3	3	4	2	3	3	3	3	3	1
Respondent 344	3	3	4	4	3	2	3	4	3	4	4	3
Respondent 345	1	5	1	2	5	3	2	2	3	1	3	4
Respondent 346	1	1	2	3	3	4	3	2	3	3	2	4
Respondent 347	2	3	3	4	4	3	2	2	5	5	2	4
Respondent 348	2	4	2	1	2	2	3	2	3	1	3	1
Respondent 349	2	1	3	3	3	3	4	3	4	3	3	2
Respondent 350	2	2	4	2	4	4	3	4	2	1	4	3
Respondent 351	2	2	3	3	3	3	4	2	3	2	3	3
Respondent 352	3	2	4	2	3	4	2	2	4	2	4	5
Respondent 353	1	3	1	3	2	2	3	3	1	3	1	3
Respondent 354	1	4	2	4	3	2	3	3	3	3	3	4
Respondent 355	2	1	2	3	2	2	2	4	2	2	4	1
Respondent 356	2	2	2	4	3	2	2	3	3	3	2	2
Respondent 357	3	3	3	4	4	3	3	5	2	2	2	2
Respondent 358	2	2	4	3	3	4	3	2	3	2	1	2
Respondent 359	2	2	1	5	4	2	4	3	4	3	2	3
Respondent 360	3	3	3	3	4	3	3	4	3	1	3	4
Respondent 361	1	3	5	4	3	5	5	1	4	2	4	1
Respondent 362	2	4	3	2	4	2	4	2	2	2	2	2
Respondent 363	2	3	4	3	3	2	3	3	3	2	1	2
Respondent 364	3	4	1	4	5	3	2	3	4	3	2	3
Respondent 365	2	1	2	1	3	4	3	4	3	1	3	4
Respondent 366	2	3	2	3	4	3	3	3	4	2	4	1
Respondent 367	3	4	2	2	2	2	5	3	2	2	1	2
Respondent 368	1	2	3	3	3	3	3	2	3	3	1	4
Respondent 369	2	2	4	2	4	4	4	3	1	4	2	1
Respondent 370	2	1	1	3	3	3	2	2	2	2	3	3
Respondent 371	3	2	2	2	3	4	3	2	1	2	2	4
Respondent 372	2	3	3	2	2	2	4	3	2	1	2	2
Respondent 373	2	4	2	3	3	2	3	3	2	2	3	4

Respondent 374	3	1	3	3	2	2	2	4	3	3	3	1
Respondent 375	1	2	4	4	3	2	2	3	3	4	4	3
Respondent 376	1	3	3	3	4	3	3	5	3	1	3	4
Respondent 377	2	2	5	5	3	4	3	2	3	3	2	4
Respondent 378	2	2	1	3	4	2	4	3	5	5	2	4
Respondent 379	3	3	2	4	4	3	3	4	3	1	3	1
Respondent 380	2	3	2	2	3	5	5	1	4	3	3	2
Respondent 381	2	4	2	3	4	2	4	2	2	1	4	3
Respondent 382	1	3	3	4	3	2	3	3	3	2	3	3
Respondent 383	2	1	4	1	5	3	2	3	4	2	4	5
Respondent 384	2	1	1	3	3	4	3	4	1	3	1	3
Respondent 385	1	2	2	2	4	3	3	3	3	3	3	4
Respondent 386	2	3	3	3	2	2	5	3	2	2	4	1
Respondent 387	2	2	2	2	3	3	3	2	3	3	2	2
Respondent 388	2	2	3	3	4	4	4	3	2	2	2	2
Respondent 389	3	3	4	4	3	3	2	2	3	2	1	2
Respondent 390	1	3	3	3	3	4	3	2	4	3	2	3
Respondent 391	1	4	4	4	2	2	4	3	3	1	3	4
Respondent 393	2	3	1	4	3	2	3	3	4	2	4	1
Respondent 394	2	5	2	3	2	2	2	2	2	2	2	2
Respondent 395	3	1	2	5	3	3	3	3	3	2	1	2
Respondent 396	2	3	2	3	4	4	4	4	4	3	2	3
Respondent 397	1	4	3	4	3	3	3	3	3	1	3	4
Respondent 398	2	1	4	2	4	3	4	3	4	2	4	1
Respondent 399	2	2	1	3	4	2	2	2	2	2	1	2
Respondent 400	3	1	3	4	3	3	2	3	3	3	1	4
Respondent 401	2	2	5	1	4	2	2	2	1	4	2	1
Respondent 402	2	3	3	3	3	3	2	3	2	2	3	3
Respondent 403	3	4	4	2	5	4	3	4	1	2	2	4
Respondent 404	1	1	1	3	3	3	4	3	2	1	2	2
Respondent 405	2	2	2	2	4	4	2	4	2	2	3	4
Respondent 406	3	3	2	3	2	2	2	2	3	3	3	1
Respondent 407	2	2	2	2	3	3	3	3	3	4	4	3
Respondent 408	2	2	3	2	4	4	4	3	3	1	3	4
Respondent 409	3	3	4	3	3	3	2	2	3	3	2	4
Respondent 410	1	3	1	3	3	4	3	2	5	5	2	4
Respondent 411	2	4	2	4	2	2	4	3	3	1	3	1
Respondent 412	2	3	3	3	3	2	3	3	4	3	3	2
Respondent 413	2	5	2	5	2	2	2	4	2	1	4	3
Respondent 414	3	1	3	3	3	2	2	3	3	2	3	3
Respondent 415	2	3	4	4	4	3	3	5	4	2	4	5
Respondent 416	2	2	3	2	3	4	3	2	1	3	1	3
Respondent 417	3	2	5	3	4	2	4	3	3	3	3	4

Response to questionnaire (25-36)

	Cow1	Cow2	Cow3	Cow4	Nat1	Nat2	Nat3	Nat4	Com1	Com2	Com3	Com4
Respondent 1	2	3	3	3	3	3	4	4	2	2	3	4
Respondent 2	2	4	2	2	4	2	4	2	2	2	4	3
Respondent 3	3	3	4	2	2	2	3	2	4	3	4	2
Respondent 4	3	3	4	4	2	3	4	3	2	3	3	3
Respondent 5	4	1	3	2	2	4	3	5	5	2	3	3
Respondent 6	3	3	4	5	2	3	4	3	3	2	4	3
Respondent 7	5	4	2	3	4	2	5	3	5	3	3	4
Respondent 8	2	2	4	3	4	3	3	3	3	2	3	3
Respondent 9	3	3	5	5	3	4	4	4	2	2	4	3
Respondent 10	4	2	4	3	2	3	4	3	3	3	3	4
Respondent 11	2	2	3	2	4	4	2	5	2	3	3	4
Respondent 12	2	3	4	3	2	3	4	3	4	4	2	3
Respondent 13	2	4	3	2	2	4	3	4	4	3	3	4
Respondent 14	2	3	4	4	3	4	2	4	3	5	4	3
Respondent 15	4	2	5	4	3	2	3	3	3	2	2	4
Respondent 16	4	3	3	3	2	3	3	5	4	3	3	4
Respondent 17	3	4	4	3	2	4	3	4	2	2	3	4
Respondent 18	2	3	4	4	3	3	3	3	3	3	3	4
Respondent 19	4	4	2	2	2	3	3	5	2	4	2	4
Respondent 20	2	3	4	3	2	4	2	4	2	3	2	3
Respondent 21	2	4	3	2	3	3	4	3	4	2	3	4
Respondent 22	3	4	2	2	3	3	4	3	5	2	4	3
Respondent 23	3	2	3	4	4	1	3	3	2	2	3	4
Respondent 24	2	3	3	5	3	3	4	3	3	3	2	5
Respondent 25	2	4	3	2	5	4	2	4	3	4	2	3
Respondent 26	3	3	3	3	2	2	4	3	2	3	4	4
Respondent 27	2	3	3	3	3	3	5	3	2	2	3	3
Respondent 28	2	4	2	2	4	2	4	4	4	4	4	2
Respondent 29	3	3	4	2	3	3	4	4	2	3	3	3
Respondent 30	3	3	4	4	4	2	4	2	5	3	3	4
Respondent 31	4	1	3	2	2	2	3	2	3	4	2	4
Respondent 32	3	3	4	5	2	3	4	3	3	2	2	3
Respondent 33	5	4	2	3	2	4	3	5	5	2	3	4
Respondent 34	2	2	4	3	2	3	4	3	3	2	4	3
Respondent 35	3	3	5	5	4	2	4	4	2	2	3	4
Respondent 36	4	2	4	3	4	3	3	3	3	4	2	5
Respondent 37	2	2	3	2	3	4	4	4	2	4	3	3
Respondent 38	2	3	4	3	2	3	4	4	4	3	4	4
Respondent 39	2	4	3	2	4	4	3	5	4	2	3	4
Respondent 40	2	3	4	4	2	3	4	3	3	4	4	2
Respondent 41	4	2	5	4	2	4	3	4	3	2	3	4
Respondent 42	4	3	3	3	3	4	2	4	4	2	4	3
Respondent 43	3	4	4	3	3	3	3	3	2	3	4	2
Respondent 44	2	3	4	4	2	3	3	5	3	3	2	3
Respondent 45	4	4	2	2	2	4	3	4	2	2	3	3
Respondent 46	2	3	4	3	3	3	4	3	2	2	4	3
Respondent 47	2	4	3	2	2	3	3	5	2	2	3	4
Respondent 48	3	4	2	2	2	4	3	4	2	2	4	3
Respondent 49	3	2	3	4	3	3	4	3	4	3	4	2
Respondent 50	2	3	3	5	3	3	4	3	2	3	3	3
Respondent 51	2	4	3	2	4	2	3	3	5	2	3	3

Respondent 52	3	3	3	3	3	3	4	3	3	2	4	3
Respondent 53	2	3	3	3	5	4	3	4	5	3	3	4
Respondent 54	2	4	2	2	2	2	4	3	3	2	3	3
Respondent 55	3	3	4	2	3	3	4	3	2	2	4	3
Respondent 56	3	3	4	4	2	3	4	4	3	3	3	4
Respondent 57	4	1	3	2	3	3	4	4	2	3	3	4
Respondent 58	3	3	4	5	4	2	4	2	4	4	2	3
Respondent 59	5	4	2	3	3	2	3	2	5	5	2	3
Respondent 60	2	2	4	3	2	3	4	3	3	3	2	4
Respondent 61	3	3	5	5	2	4	3	5	3	5	3	3
Respondent 62	4	2	4	3	2	3	4	3	3	3	2	3
Respondent 63	2	2	3	2	3	2	5	3	4	2	2	4
Respondent 64	2	3	4	3	4	2	3	3	3	3	3	3
Respondent 65	2	4	3	2	3	4	4	4	5	2	3	3
Respondent 66	2	3	4	4	2	3	3	3	3	4	4	2
Respondent 67	4	2	5	4	4	4	2	5	4	4	3	3
Respondent 68	4	3	3	3	3	3	3	3	4	3	5	4
Respondent 69	3	4	4	3	3	3	4	4	3	3	2	2
Respondent 70	2	3	4	4	4	2	4	2	5	4	3	3
Respondent 71	4	4	2	2	2	2	3	2	4	2	2	3
Respondent 72	2	3	4	3	2	3	4	3	3	3	3	3
Respondent 73	2	4	3	2	2	4	3	5	5	2	4	2
Respondent 74	3	4	2	2	2	3	4	3	4	2	3	2
Respondent 75	3	2	3	4	4	2	5	3	3	4	2	3
Respondent 76	2	3	3	5	4	3	3	3	3	5	2	4
Respondent 77	2	4	3	2	3	4	4	4	3	2	2	3
Respondent 78	3	3	3	3	2	3	4	3	3	3	3	2
Respondent 79	2	3	3	3	4	4	2	5	4	3	4	2
Respondent 80	2	4	2	2	2	3	4	3	3	2	3	4
Respondent 81	3	3	4	2	2	4	3	4	3	2	2	3
Respondent 82	3	3	4	4	3	4	2	4	4	4	4	4
Respondent 83	4	1	3	2	3	2	3	3	4	2	3	3
Respondent 84	3	3	4	5	2	3	3	5	2	5	3	3
Respondent 85	5	4	2	3	2	4	3	4	2	3	4	2
Respondent 86	2	2	4	3	3	3	3	3	3	3	2	2
Respondent 87	3	3	5	5	2	3	3	5	5	5	2	3
Respondent 88	4	2	4	3	2	4	2	4	3	3	2	4
Respondent 89	2	2	3	2	3	3	4	3	4	2	2	3
Respondent 90	2	3	4	3	3	3	4	3	3	3	4	2
Respondent 91	2	4	3	2	4	1	3	3	4	2	4	3
Respondent 92	2	3	4	4	3	3	4	3	4	4	3	4
Respondent 93	4	2	5	4	5	4	2	4	5	4	2	3
Respondent 94	4	3	3	3	2	2	4	3	3	3	4	4
Respondent 95	3	4	4	3	3	3	5	3	4	3	2	3
Respondent 96	2	3	4	4	4	2	4	4	4	4	2	4
Respondent 97	4	4	2	2	3	3	4	4	3	2	3	4
Respondent 98	2	3	4	3	4	2	4	2	5	3	3	2
Respondent 99	2	4	3	2	2	2	3	2	4	2	2	3
Respondent 100	3	4	2	2	2	3	4	3	3	2	2	4
Respondent 101	3	2	3	4	2	4	3	5	5	2	2	3
Respondent 102	2	3	3	5	2	3	4	3	4	2	2	4
Respondent 103	2	4	3	2	4	2	4	4	3	4	3	4
Respondent 104	3	3	3	3	4	3	3	3	3	2	3	3
Respondent 105	2	3	3	3	3	4	4	4	3	5	2	3

Respondent 106	2	4	2	2	2	3	4	4	3	3	2	4
Respondent 107	3	3	4	2	4	4	3	5	4	5	3	3
Respondent 108	3	3	4	4	2	3	4	3	3	3	2	3
Respondent 109	4	1	3	2	2	4	3	4	3	2	2	4
Respondent 110	3	3	4	5	3	4	2	4	4	3	3	3
Respondent 111	5	4	2	3	3	3	3	3	4	2	3	3
Respondent 112	2	2	4	3	2	3	3	5	2	4	4	2
Respondent 113	4	2	4	3	2	4	3	4	5	5	2	3
Respondent 114	2	2	3	2	3	3	4	3	3	3	2	4
Respondent 115	2	3	4	3	2	3	3	5	3	5	3	3
Respondent 116	2	4	3	2	2	4	3	4	3	3	2	3
Respondent 117	2	3	4	4	3	3	4	3	4	2	2	4
Respondent 118	4	2	5	4	3	3	4	3	3	3	3	3
Respondent 119	4	3	3	3	4	2	3	3	5	2	3	3
Respondent 120	3	4	4	3	3	3	4	3	3	4	4	2
Respondent 121	2	3	4	4	5	4	3	4	4	4	3	3
Respondent 122	4	4	2	2	2	2	4	3	4	3	5	4
Respondent 123	2	3	4	3	3	3	4	3	3	3	2	2
Respondent 124	2	4	3	2	2	3	4	4	5	4	3	3
Respondent 125	3	4	2	2	3	3	4	4	4	2	2	3
Respondent 126	3	2	3	4	4	2	4	2	3	3	3	3
Respondent 127	2	3	3	5	3	2	3	2	5	2	4	2
Respondent 128	2	4	3	2	2	3	4	3	4	2	3	2
Respondent 129	3	3	3	3	2	4	3	5	3	4	2	3
Respondent 130	2	3	3	3	2	3	4	3	3	5	2	4
Respondent 131	2	4	2	2	3	2	5	3	3	2	2	3
Respondent 132	3	3	4	2	4	2	3	3	3	3	3	2
Respondent 133	3	3	4	4	3	4	4	4	4	3	4	2
Respondent 134	4	1	3	2	2	3	3	3	3	2	3	4
Respondent 135	3	3	4	5	4	4	2	5	3	2	2	3
Respondent 136	5	4	2	3	3	3	3	3	4	4	4	4
Respondent 137	2	2	4	3	3	3	4	4	4	2	3	3
Respondent 138	3	3	5	5	4	2	4	2	2	5	3	3
Respondent 139	4	2	4	3	2	2	3	2	2	3	4	2
Respondent 140	2	2	3	2	2	3	4	3	3	3	2	2
Respondent 141	2	3	4	3	2	4	3	5	5	5	2	3
Respondent 142	2	4	3	2	2	3	4	3	3	3	2	4
Respondent 143	2	3	4	4	4	2	5	3	4	2	2	3
Respondent 144	4	2	5	4	4	3	3	3	3	3	4	2
Respondent 145	4	3	3	3	3	4	4	4	4	2	4	3
Respondent 146	3	4	4	3	2	3	4	3	4	4	3	4
Respondent 147	2	3	4	4	4	4	2	5	5	4	2	3
Respondent 148	4	4	2	2	2	3	4	3	3	3	4	4
Respondent 149	2	3	4	3	2	4	3	4	4	3	2	3
Respondent 150	2	4	3	2	3	4	2	4	4	4	2	4
Respondent 151	3	4	2	2	3	2	3	3	3	2	3	4
Respondent 152	3	2	3	4	2	3	3	5	5	3	3	2
Respondent 153	3	3	3	3	2	4	3	4	4	2	2	3
Respondent 154	2	3	3	3	3	3	3	3	3	2	2	4
Respondent 155	2	4	2	2	2	3	3	5	5	2	2	3
Respondent 156	3	3	4	2	2	4	2	4	4	2	2	4
Respondent 157	3	3	4	4	3	3	4	3	3	4	3	4
Respondent 158	3	2	3	2	3	3	4	3	3	2	3	3
Respondent 159	3	3	4	5	4	1	3	3	3	5	2	3

Respondent 160	4	2	4	3	3	3	4	3	3	3	2	4
Respondent 161	3	3	4	5	5	4	2	4	4	5	3	3
Respondent 162	4	2	4	3	2	2	4	3	3	3	2	3
Respondent 163	2	2	3	2	3	3	5	3	3	2	2	4
Respondent 164	2	3	4	3	4	2	4	4	4	3	3	3
Respondent 165	2	4	3	2	3	3	4	4	4	2	3	3
Respondent 166	2	3	4	4	4	2	4	2	2	4	4	2
Respondent 167	4	2	5	4	2	2	3	2	4	2	5	3
Respondent 168	4	3	3	3	2	3	4	3	3	3	3	4
Respondent 169	3	4	4	3	2	4	3	5	3	4	4	4
Respondent 170	2	3	4	4	2	3	4	3	2	4	2	2
Respondent 171	4	4	2	2	4	2	4	4	2	3	2	2
Respondent 172	2	3	4	3	4	3	3	3	3	4	3	3
Respondent 173	2	4	3	2	3	4	4	4	4	3	5	5
Respondent 174	3	4	2	2	2	3	4	4	3	4	3	3
Respondent 175	3	2	3	4	4	4	3	5	2	5	3	4
Respondent 176	2	3	3	5	2	3	4	3	3	3	3	3
Respondent 177	2	4	3	2	2	4	3	4	4	4	4	4
Respondent 178	3	3	3	3	3	4	2	4	3	4	3	4
Respondent 179	2	3	3	3	3	3	3	3	4	2	5	5
Respondent 180	2	4	2	2	2	3	3	5	3	4	3	3
Respondent 181	3	3	4	2	2	4	3	4	4	3	4	4
Respondent 182	3	3	4	4	3	3	4	3	4	2	4	4
Respondent 183	4	1	3	2	2	3	3	5	2	3	3	3
Respondent 184	3	3	4	5	2	4	3	4	3	3	5	5
Respondent 185	5	4	2	3	3	3	4	3	4	3	4	4
Respondent 186	2	2	4	3	3	3	4	3	3	3	3	3
Respondent 187	3	3	5	5	4	2	3	3	3	3	5	5
Respondent 188	4	2	4	3	3	3	4	3	4	2	4	4
Respondent 189	2	2	3	2	5	4	3	4	3	4	3	3
Respondent 190	2	3	4	3	2	2	4	3	3	4	3	3
Respondent 191	2	4	3	2	3	3	4	3	1	3	3	3
Respondent 192	2	3	4	4	2	3	4	4	3	4	3	3
Respondent 193	4	2	5	4	3	3	4	4	4	2	4	4
Respondent 194	4	3	3	3	4	2	4	2	2	4	3	3
Respondent 195	3	4	4	3	3	2	3	2	3	5	3	3
Respondent 196	2	3	4	4	2	3	4	3	2	4	4	4
Respondent 197	4	4	2	2	2	4	3	5	3	4	4	4
Respondent 198	2	3	4	3	2	3	4	3	2	4	2	2
Respondent 199	2	4	3	2	3	2	5	3	4	2	5	3
Respondent 200	3	4	2	2	4	2	3	3	3	3	3	4
Respondent 201	3	2	3	4	3	4	4	4	3	4	4	4
Respondent 202	2	3	3	5	2	3	3	3	2	4	2	2
Respondent 203	2	4	3	2	4	4	2	5	2	3	2	2
Respondent 204	3	3	3	3	3	3	3	3	3	4	3	3
Respondent 205	2	3	3	3	3	3	4	4	4	3	5	5
Respondent 206	2	4	2	2	4	2	4	2	3	4	3	3
Respondent 207	3	3	4	2	2	2	3	2	2	5	3	4
Respondent 208	3	3	4	4	2	3	4	3	3	3	3	3
Respondent 209	3	2	3	2	2	4	3	5	4	4	4	4
Respondent 210	3	3	4	5	2	3	4	3	3	4	3	4
Respondent 211	4	2	4	3	4	2	5	3	4	2	5	5
Respondent 212	2	2	3	2	4	3	3	3	3	4	3	3
Respondent 213	2	3	4	3	3	4	4	4	4	3	4	4

Respondent 214	2	4	3	2	2	3	4	3	4	2	4	4
Respondent 215	3	2	3	4	4	4	2	5	2	3	3	3
Respondent 216	4	2	5	4	2	3	4	3	3	3	5	5
Respondent 217	4	3	3	3	2	4	3	4	4	3	4	4
Respondent 218	3	4	4	3	3	4	2	4	3	3	3	3
Respondent 219	2	3	4	4	3	2	3	3	3	3	5	5
Respondent 220	4	4	2	2	2	3	3	5	4	2	4	4
Respondent 221	2	3	4	3	2	4	3	4	3	4	3	4
Respondent 222	2	4	3	2	3	3	3	3	3	4	3	3
Respondent 223	3	4	2	2	2	3	3	5	1	3	3	3
Respondent 224	3	2	3	4	2	4	2	4	3	4	3	4
Respondent 225	2	3	3	5	3	3	4	3	4	2	4	4
Respondent 226	2	4	3	2	3	3	4	3	2	4	3	3
Respondent 227	3	3	3	3	4	1	3	3	3	5	3	3
Respondent 228	4	2	4	3	3	3	4	3	2	4	4	4
Respondent 229	2	2	3	2	5	4	2	4	3	4	4	4
Respondent 230	2	3	4	3	2	2	4	3	2	4	2	2
Respondent 231	2	4	3	2	3	3	5	3	4	2	5	3
Respondent 232	2	3	4	4	4	2	4	4	3	3	3	4
Respondent 233	4	2	5	4	3	3	4	4	3	4	4	4
Respondent 234	4	3	3	3	4	2	4	2	2	4	2	2
Respondent 235	3	4	4	3	2	2	3	2	2	3	2	2
Respondent 236	2	3	4	4	2	3	4	3	3	4	3	3
Respondent 237	4	4	2	2	2	4	3	5	4	3	5	5
Respondent 238	2	3	4	3	2	3	4	3	3	4	3	3
Respondent 239	2	4	3	2	4	2	4	4	2	5	3	4
Respondent 240	3	4	2	2	4	3	3	3	3	3	3	3
Respondent 241	3	2	3	4	3	4	4	4	4	4	4	4
Respondent 242	3	3	3	3	2	3	4	4	3	4	3	4
Respondent 243	2	3	3	3	4	4	3	5	4	2	5	5
Respondent 244	2	4	2	2	2	3	4	3	3	4	3	3
Respondent 245	3	3	4	2	2	4	3	4	4	3	4	4
Respondent 246	3	3	4	4	3	4	2	4	4	2	4	4
Respondent 247	3	2	3	2	3	3	3	3	2	3	3	3
Respondent 248	3	3	4	5	2	3	3	5	3	3	5	5
Respondent 249	4	2	4	3	2	4	3	4	4	3	4	4
Respondent 250	3	3	4	5	3	3	4	3	3	3	3	3
Respondent 251	4	2	4	3	2	3	3	5	3	3	5	5
Respondent 252	2	2	3	2	2	4	3	4	4	2	4	4
Respondent 253	2	3	4	3	3	3	4	3	3	4	3	3
Respondent 254	2	4	3	2	3	3	4	3	3	4	3	3
Respondent 255	2	3	4	4	4	2	3	3	1	3	3	3
Respondent 256	4	2	5	4	3	3	4	3	3	4	3	3
Respondent 257	4	3	3	3	5	4	3	4	4	2	4	4
Respondent 258	3	4	4	3	2	2	4	3	2	4	3	3
Respondent 259	2	3	4	4	3	3	4	3	3	5	3	3
Respondent 260	4	4	2	2	2	3	4	4	2	4	4	4
Respondent 261	2	3	4	3	3	3	4	4	3	4	4	4
Respondent 262	2	4	3	2	4	2	4	2	2	4	2	2
Respondent 263	3	4	2	2	3	2	3	2	4	2	5	3
Respondent 264	3	2	3	4	2	3	4	3	3	3	3	4
Respondent 265	2	3	3	5	2	4	3	5	3	4	4	4
Respondent 266	2	4	3	2	2	3	4	3	2	4	2	3
Respondent 267	3	3	3	3	3	2	5	3	2	3	2	2

Respondent 268	2	3	3	3	4	2	3	3	3	4	3	3
Respondent 269	2	4	2	2	3	4	4	4	4	3	5	5
Respondent 270	3	3	4	2	2	3	3	3	3	4	3	3
Respondent 271	3	3	4	4	4	4	2	5	2	5	3	4
Respondent 272	4	1	3	2	3	3	3	3	4	4	4	4
Respondent 273	3	3	4	5	3	3	4	4	4	3	4	3
Respondent 274	5	4	2	3	4	2	4	2	5	4	2	5
Respondent 275	2	2	4	3	2	2	3	2	3	3	4	3
Respondent 276	3	3	5	5	2	3	4	3	4	4	3	4
Respondent 277	4	2	4	3	2	4	3	5	4	4	2	4
Respondent 278	2	2	3	2	2	3	4	3	3	2	3	3
Respondent 279	2	3	4	3	4	2	5	3	5	3	3	5
Respondent 280	2	4	3	2	4	3	3	3	4	4	3	4
Respondent 281	2	3	4	4	3	4	4	4	3	3	3	3
Respondent 282	4	2	5	4	2	3	4	3	5	3	3	5
Respondent 283	4	3	3	3	4	4	2	5	4	4	2	4
Respondent 284	3	4	4	3	2	3	4	3	3	3	4	3
Respondent 285	2	3	4	4	2	4	3	4	3	3	4	3
Respondent 286	4	4	2	2	3	4	2	4	3	1	3	3
Respondent 287	2	3	4	3	3	2	3	3	3	3	4	3
Respondent 288	2	4	3	2	2	3	3	5	4	4	2	4
Respondent 289	3	4	2	2	2	4	3	4	3	2	4	3
Respondent 290	3	2	3	4	3	3	3	3	3	3	5	3
Respondent 291	2	3	3	5	2	3	3	5	4	2	4	4
Respondent 292	2	4	3	2	2	4	2	4	4	3	4	4
Respondent 293	3	3	3	3	3	3	4	3	2	2	4	4
Respondent 294	2	3	3	3	3	3	4	3	2	4	2	5
Respondent 295	2	4	2	2	4	3	3	3	3	3	3	3
Respondent 296	3	3	4	2	3	3	4	3	5	3	4	4
Respondent 297	3	3	4	4	5	4	2	4	3	2	4	2
Respondent 298	3	2	3	2	2	2	4	3	3	2	3	2
Respondent 299	3	3	4	5	3	3	5	3	3	3	4	3
Respondent 300	4	2	4	3	4	2	4	4	4	4	3	5
Respondent 301	2	2	3	2	3	3	4	4	3	3	4	4
Respondent 302	2	3	4	3	4	2	4	2	5	2	5	3
Respondent 303	2	4	3	2	2	2	3	2	4	4	4	4
Respondent 304	3	2	3	4	2	3	4	3	4	3	4	3
Respondent 305	4	2	5	4	2	4	3	5	5	4	2	5
Respondent 306	4	3	3	3	2	3	4	3	3	3	4	3
Respondent 307	3	4	4	3	4	2	4	4	4	4	3	4
Respondent 308	4	2	4	3	4	3	3	3	4	4	2	4
Respondent 309	2	2	3	2	3	4	4	4	3	2	3	3
Respondent 310	2	3	4	3	2	3	4	4	5	3	3	5
Respondent 311	2	4	3	2	4	4	3	5	4	4	3	4
Respondent 312	2	3	4	4	2	3	4	3	3	3	3	3
Respondent 313	4	2	5	4	2	4	3	4	5	3	3	5
Respondent 314	4	3	3	3	3	4	2	4	4	4	2	4
Respondent 315	3	4	4	3	3	3	3	3	3	3	4	3
Respondent 316	2	3	4	4	2	3	3	5	3	3	4	4
Respondent 317	4	4	2	2	2	4	3	4	3	1	3	3
Respondent 318	2	3	4	3	3	3	4	3	3	3	4	3
Respondent 319	2	4	3	2	2	3	3	5	4	4	2	4
Respondent 320	3	4	2	2	2	4	3	4	3	2	4	3
Respondent 321	3	2	3	4	3	3	4	3	3	3	5	3

Respondent 322	3	3	3	3	3	3	4	3	4	2	4	4
Respondent 323	2	3	3	3	4	2	3	3	4	3	4	4
Respondent 324	2	4	2	2	3	3	4	3	2	2	4	2
Respondent 325	3	3	4	2	5	4	3	4	2	4	2	5
Respondent 326	3	3	4	4	2	2	4	3	3	3	3	3
Respondent 327	3	2	3	2	3	3	4	3	5	3	4	4
Respondent 328	3	3	4	5	2	3	4	4	3	2	4	2
Respondent 329	4	2	4	3	3	3	4	4	3	2	3	5
Respondent 330	3	3	4	5	4	2	4	2	3	3	4	3
Respondent 331	4	2	4	3	3	2	3	2	4	4	3	5
Respondent 332	2	2	3	2	2	3	4	3	3	3	4	3
Respondent 333	2	3	4	3	2	4	3	5	5	2	5	3
Respondent 334	2	4	3	2	2	3	4	3	3	4	4	4
Respondent 335	2	3	4	4	3	2	5	3	4	4	3	4
Respondent 336	4	2	5	4	4	2	3	3	2	5	4	2
Respondent 337	4	3	3	3	3	4	4	4	2	3	3	4
Respondent 338	3	4	4	3	2	3	3	3	3	4	4	3
Respondent 339	2	3	4	4	4	4	2	5	5	4	4	4
Respondent 340	4	4	2	2	3	3	3	3	3	3	2	5
Respondent 341	2	3	4	3	3	3	4	4	3	5	3	5
Respondent 342	2	4	3	2	4	2	4	2	3	4	4	5
Respondent 343	3	4	2	2	2	2	3	2	4	3	3	3
Respondent 344	3	2	3	4	2	3	4	3	3	5	3	3
Respondent 345	2	3	3	5	2	4	3	5	5	4	4	2
Respondent 346	2	4	3	2	2	3	4	3	3	3	3	4
Respondent 347	3	3	3	3	4	2	5	3	4	3	3	4
Respondent 348	2	3	3	3	4	3	3	3	4	3	1	3
Respondent 349	2	4	2	2	3	4	4	4	3	3	3	4
Respondent 350	3	3	4	2	2	3	4	3	5	4	4	2
Respondent 351	3	3	4	4	4	4	2	5	4	3	2	4
Respondent 352	4	1	3	2	2	3	4	3	3	3	3	5
Respondent 353	3	3	4	5	2	4	3	4	5	4	2	4
Respondent 354	5	4	2	3	3	4	2	4	4	4	3	4
Respondent 355	2	2	4	3	3	2	3	3	3	2	2	4
Respondent 356	3	3	5	5	2	3	3	5	3	2	4	2
Respondent 357	4	2	4	3	2	4	3	4	3	3	3	5
Respondent 358	2	2	3	2	3	3	3	3	3	5	3	4
Respondent 359	2	3	4	3	2	3	3	5	4	3	2	4
Respondent 360	2	4	3	2	2	4	2	4	3	3	2	3
Respondent 361	2	3	4	4	3	3	4	3	3	3	3	4
Respondent 362	4	2	5	4	3	3	4	3	4	4	4	3
Respondent 363	4	3	3	3	4	2	3	3	4	3	3	4
Respondent 364	3	4	4	3	3	3	4	3	2	5	2	5
Respondent 365	2	3	4	4	5	4	2	4	2	4	4	4
Respondent 366	4	4	2	2	2	2	4	3	4	3	4	4
Respondent 367	2	3	4	3	3	3	5	3	2	2	4	2
Respondent 368	2	4	3	2	4	2	4	4	2	4	2	5
Respondent 369	3	4	2	2	3	3	4	4	3	3	3	3
Respondent 370	3	2	3	4	4	2	4	2	5	3	4	4
Respondent 371	2	3	3	5	2	2	3	2	3	2	4	2
Respondent 372	2	4	3	2	2	3	4	3	3	2	3	4
Respondent 373	3	3	3	3	2	4	3	5	3	3	4	3
Respondent 374	2	3	3	3	2	3	4	3	4	4	3	5
Respondent 375	2	4	2	2	4	2	4	4	3	3	4	5

Respondent 376	3	3	4	2	4	3	3	3	5	2	5	3
Respondent 377	3	3	4	4	3	4	4	4	3	4	4	4
Respondent 378	3	2	3	2	2	3	4	4	4	4	3	4
Respondent 379	3	3	4	5	4	4	3	5	2	5	4	2
Respondent 380	4	2	4	3	2	3	4	3	2	3	3	4
Respondent 381	2	2	3	2	2	4	3	4	3	4	4	3
Respondent 382	2	3	4	3	3	4	2	4	5	4	4	4
Respondent 383	2	4	3	2	3	3	3	3	3	3	2	3
Respondent 384	3	2	3	4	2	3	3	5	3	5	3	5
Respondent 385	4	2	5	4	2	4	3	4	3	4	4	3
Respondent 386	4	3	3	3	3	3	4	3	4	3	3	5
Respondent 387	3	4	4	3	2	3	3	5	3	5	3	3
Respondent 388	4	2	4	3	2	4	3	4	5	4	4	5
Respondent 389	2	2	3	2	3	3	4	3	3	3	3	4
Respondent 390	2	3	4	3	3	3	4	3	4	3	3	4
Respondent 391	2	4	3	2	4	2	3	3	4	3	1	3
Respondent 393	2	3	4	4	3	3	4	3	3	3	3	4
Respondent 394	4	2	5	4	5	4	3	4	5	4	4	3
Respondent 395	4	3	3	3	2	2	4	3	4	3	2	4
Respondent 396	3	4	4	3	3	3	4	3	3	3	3	5
Respondent 397	2	3	4	4	2	3	4	4	5	4	2	4
Respondent 398	4	4	2	2	3	3	4	4	4	4	3	4
Respondent 399	2	3	4	3	4	2	4	2	3	2	2	4
Respondent 400	2	4	3	2	3	2	3	2	3	2	4	2
Respondent 401	3	4	2	2	2	3	4	3	3	3	3	3
Respondent 402	3	2	3	4	2	4	3	5	3	5	3	4
Respondent 403	3	3	3	3	2	3	4	3	4	3	2	4
Respondent 404	2	3	3	3	3	2	5	3	3	3	2	3
Respondent 405	2	4	2	2	4	2	3	3	3	3	3	4
Respondent 406	3	3	4	2	3	4	4	4	4	4	4	3
Respondent 407	3	3	4	4	2	3	3	3	4	3	3	4
Respondent 408	3	2	3	2	4	4	2	5	2	5	2	5
Respondent 409	3	3	4	5	3	3	3	3	2	4	4	4
Respondent 410	4	2	4	3	3	3	4	4	4	3	4	5
Respondent 411	3	3	4	5	4	2	4	2	2	2	4	2
Respondent 412	4	2	4	3	2	2	3	2	2	4	2	5
Respondent 413	2	2	3	2	2	3	4	3	3	3	3	3
Respondent 414	2	3	4	3	2	4	3	5	5	3	4	4
Respondent 415	2	4	3	2	2	3	4	3	3	2	4	3
Respondent 416	2	3	4	4	4	2	5	3	3	2	3	2
Respondent 417	4	2	5	4	4	3	3	3	3	3	4	3

Response to questionnaire (37-39)

Response of their turnover intention

[1=Very unlikely, 2=Unlikely, 3=Likely, 4=Very likely]

	Opinion
Respondent 1	1
Respondent 2	3
Respondent 3	1
Respondent 4	2
Respondent 5	3
Respondent 6	1
Respondent 7	3
Respondent 8	3
Respondent 9	1
Respondent 10	2
Respondent 11	4
Respondent 12	1
Respondent 13	3
Respondent 14	4
Respondent 15	3
Respondent 16	3
Respondent 17	4
Respondent 18	3
Respondent 19	2
Respondent 20	3
Respondent 21	4
Respondent 22	1
Respondent 23	3
Respondent 24	2
Respondent 25	4
Respondent 26	3
Respondent 27	3
Respondent 28	3
Respondent 29	2
Respondent 30	3
Respondent 31	4
Respondent 32	3
Respondent 33	1
Respondent 34	2
Respondent 35	3
Respondent 36	3
Respondent 37	1
Respondent 38	4
Respondent 39	3
Respondent 40	2
Respondent 41	4
Respondent 42	1
Respondent 43	3
Respondent 44	4
Respondent 45	2

Respondent 46	3
Respondent 47	1
Respondent 48	4
Respondent 49	4
Respondent 50	3
Respondent 51	2
Respondent 52	1
Respondent 53	3
Respondent 54	4
Respondent 55	3
Respondent 56	2
Respondent 57	3
Respondent 58	4
Respondent 59	1
Respondent 60	3
Respondent 61	4
Respondent 62	3
Respondent 63	1
Respondent 64	2
Respondent 65	4
Respondent 66	4
Respondent 67	1
Respondent 68	3
Respondent 69	4
Respondent 70	4
Respondent 71	1
Respondent 72	4
Respondent 73	3
Respondent 74	1
Respondent 75	4
Respondent 76	1
Respondent 77	1
Respondent 78	4
Respondent 79	3
Respondent 80	4
Respondent 81	1
Respondent 82	3
Respondent 83	4
Respondent 84	2
Respondent 85	3
Respondent 86	4
Respondent 87	4
Respondent 88	1
Respondent 89	4
Respondent 90	1
Respondent 91	3
Respondent 92	4
Respondent 93	4
Respondent 94	1
Respondent 95	1
Respondent 96	4
Respondent 97	1
Respondent 98	4
Respondent 99	2

Respondent 100	4
Respondent 101	1
Respondent 102	3
Respondent 103	4
Respondent 104	4
Respondent 105	1
Respondent 106	3
Respondent 107	4
Respondent 108	3
Respondent 109	1
Respondent 110	4
Respondent 111	3
Respondent 112	1
Respondent 113	4
Respondent 114	3
Respondent 115	1
Respondent 116	4
Respondent 117	3
Respondent 118	1
Respondent 119	3
Respondent 120	2
Respondent 121	3
Respondent 122	4
Respondent 123	1
Respondent 124	3
Respondent 125	4
Respondent 126	3
Respondent 127	2
Respondent 128	4
Respondent 129	3
Respondent 130	2
Respondent 131	4
Respondent 132	1
Respondent 133	1
Respondent 134	3
Respondent 135	4
Respondent 136	1
Respondent 137	3
Respondent 138	4
Respondent 139	1
Respondent 140	3
Respondent 141	4
Respondent 142	1
Respondent 143	3
Respondent 144	4
Respondent 145	1
Respondent 146	3
Respondent 147	1
Respondent 148	3
Respondent 149	4
Respondent 150	1
Respondent 151	3
Respondent 152	4
Respondent 153	1

Respondent 154	3
Respondent 155	4
Respondent 156	3
Respondent 157	1
Respondent 158	3
Respondent 159	4
Respondent 160	1
Respondent 161	3
Respondent 162	4
Respondent 163	2
Respondent 164	1
Respondent 165	3
Respondent 166	3
Respondent 167	1
Respondent 168	3
Respondent 169	4
Respondent 170	3
Respondent 171	1
Respondent 172	3
Respondent 173	2
Respondent 174	1
Respondent 175	3
Respondent 176	4
Respondent 177	1
Respondent 178	3
Respondent 179	4
Respondent 180	2
Respondent 181	1
Respondent 182	3
Respondent 183	4
Respondent 184	3
Respondent 185	4
Respondent 186	2
Respondent 187	1
Respondent 188	3
Respondent 189	3
Respondent 190	4
Respondent 191	2
Respondent 192	1
Respondent 193	3
Respondent 194	4
Respondent 195	3
Respondent 196	1
Respondent 197	2
Respondent 198	3
Respondent 199	4
Respondent 200	4
Respondent 201	2
Respondent 202	1
Respondent 203	3
Respondent 204	4
Respondent 205	3
Respondent 206	1
Respondent 207	4

Respondent 208	3
Respondent 209	4
Respondent 210	4
Respondent 211	1
Respondent 212	3
Respondent 213	3
Respondent 214	4
Respondent 215	3
Respondent 216	1
Respondent 217	3
Respondent 218	3
Respondent 219	1
Respondent 220	4
Respondent 221	4
Respondent 222	3
Respondent 223	1
Respondent 224	3
Respondent 225	4
Respondent 226	3
Respondent 227	1
Respondent 228	3
Respondent 229	4
Respondent 230	4
Respondent 231	1
Respondent 232	3
Respondent 233	4
Respondent 234	4
Respondent 235	1
Respondent 236	3
Respondent 237	2
Respondent 238	4
Respondent 239	1
Respondent 240	4
Respondent 241	2
Respondent 242	4
Respondent 243	1
Respondent 244	3
Respondent 245	4
Respondent 246	1
Respondent 247	3
Respondent 248	2
Respondent 249	3
Respondent 250	4
Respondent 251	1
Respondent 252	3
Respondent 253	4
Respondent 254	3
Respondent 255	4
Respondent 256	1
Respondent 257	3
Respondent 258	2
Respondent 259	4
Respondent 260	4
Respondent 261	1

Respondent 262	2
Respondent 263	4
Respondent 264	3
Respondent 265	4
Respondent 266	1
Respondent 267	3
Respondent 268	4
Respondent 269	3
Respondent 270	4
Respondent 271	1
Respondent 272	2
Respondent 273	3
Respondent 274	4
Respondent 275	1
Respondent 276	2
Respondent 277	3
Respondent 278	4
Respondent 279	3
Respondent 280	1
Respondent 281	2
Respondent 282	3
Respondent 283	4
Respondent 284	4
Respondent 285	2
Respondent 286	4
Respondent 287	2
Respondent 288	3
Respondent 289	4
Respondent 290	2
Respondent 291	3
Respondent 292	4
Respondent 293	3
Respondent 294	1
Respondent 295	3
Respondent 296	3
Respondent 297	4
Respondent 298	2
Respondent 299	3
Respondent 300	4
Respondent 301	1
Respondent 302	4
Respondent 303	3
Respondent 304	2
Respondent 305	3
Respondent 306	3
Respondent 307	1
Respondent 308	2
Respondent 309	4
Respondent 310	2
Respondent 311	2
Respondent 312	3
Respondent 313	2
Respondent 314	3
Respondent 315	4

Respondent 316	1
Respondent 317	2
Respondent 318	3
Respondent 319	2
Respondent 320	3
Respondent 321	1
Respondent 322	4
Respondent 323	2
Respondent 324	2
Respondent 325	3
Respondent 326	1
Respondent 327	3
Respondent 328	3
Respondent 329	2
Respondent 330	2
Respondent 331	1
Respondent 332	2
Respondent 333	3
Respondent 334	3
Respondent 335	2
Respondent 336	1
Respondent 337	3
Respondent 338	2
Respondent 339	4
Respondent 340	2
Respondent 341	1
Respondent 342	3
Respondent 343	2
Respondent 344	3
Respondent 345	1
Respondent 346	4
Respondent 347	2
Respondent 348	2
Respondent 349	1
Respondent 350	3
Respondent 351	3
Respondent 352	2
Respondent 353	1
Respondent 354	3
Respondent 355	4
Respondent 356	2
Respondent 357	2
Respondent 358	3
Respondent 359	3
Respondent 360	2
Respondent 361	3
Respondent 362	3
Respondent 363	2
Respondent 364	1
Respondent 365	3
Respondent 366	2
Respondent 367	3
Respondent 368	3
Respondent 369	2

Respondent 370	1
Respondent 371	3
Respondent 372	3
Respondent 373	2
Respondent 374	2
Respondent 375	2
Respondent 376	3
Respondent 377	2
Respondent 378	3
Respondent 379	1
Respondent 380	4
Respondent 381	2
Respondent 382	3
Respondent 383	1
Respondent 384	3
Respondent 385	3
Respondent 386	2
Respondent 387	3
Respondent 388	3
Respondent 389	1
Respondent 390	3
Respondent 391	2
Respondent 393	3
Respondent 394	2
Respondent 395	1
Respondent 396	3
Respondent 397	2
Respondent 398	4
Respondent 399	1
Respondent 400	3
Respondent 401	3
Respondent 402	4
Respondent 403	1
Respondent 404	3
Respondent 405	4
Respondent 406	3
Respondent 407	1
Respondent 408	3
Respondent 409	3
Respondent 410	2
Respondent 411	1
Respondent 412	3
Respondent 413	2
Respondent 414	3
Respondent 415	3
Respondent 416	1
Respondent 417	3
